# Retrograde Mitochondrial Transport Is Essential for Organelle Distribution and Health in Zebrafish Neurons

**DOI:** 10.1523/JNEUROSCI.1316-20.2020

**Published:** 2021-02-17

**Authors:** Amrita Mandal, Hiu-Tung C. Wong, Katherine Pinter, Natalie Mosqueda, Alisha Beirl, Richa Madan Lomash, Sehoon Won, Katie S. Kindt, Catherine M. Drerup

**Affiliations:** ^1^Unit on Neuronal Cell Biology, National Institute of Child Health and Human Development, National Institutes of Health, Bethesda, Maryland 20892; ^2^Section on Sensory Cell Development and Function, National Institute of Deafness and other Communication Disorders, National Institutes of Health, Bethesda, Maryland 20892; ^3^Receptor Biology Section, National Institute of Neurological Disorders and Stroke, National Institutes of Health, Bethesda, Maryland 20892

**Keywords:** Actr10, axonal transport, cytoplasmic dynein, dynactin, mitochondria, retrograde transport

## Abstract

In neurons, mitochondria are transported by molecular motors throughout the cell to form and maintain functional neural connections. These organelles have many critical functions in neurons and are of high interest as their dysfunction is associated with disease. While the mechanics and impact of anterograde mitochondrial movement toward axon terminals are beginning to be understood, the frequency and function of retrograde (cell body directed) mitochondrial transport in neurons are still largely unexplored. While existing evidence indicates that some mitochondria are retrogradely transported for degradation in the cell body, the precise impact of disrupting retrograde transport on the organelles and the axon was unknown. Using long-term, *in vivo* imaging, we examined mitochondrial motility in zebrafish sensory and motor axons. We show that retrograde transport of mitochondria from axon terminals allows replacement of the axon terminal population within a day. By tracking these organelles, we show that not all mitochondria that leave the axon terminal are degraded; rather, they persist over several days. Disrupting retrograde mitochondrial flux in neurons leads to accumulation of aged organelles in axon terminals and loss of cell body mitochondria. Assays of neural circuit activity demonstrated that disrupting mitochondrial transport and function has no effect on sensory axon terminal activity but does negatively impact motor neuron axons. Taken together, our work supports a previously unappreciated role for retrograde mitochondrial transport in the maintenance of a homeostatic distribution of mitochondria in neurons and illustrates the downstream effects of disrupting this process on sensory and motor circuits.

**SIGNIFICANCE STATEMENT** Disrupted mitochondrial transport has been linked to neurodegenerative disease. Retrograde transport of this organelle has been implicated in turnover of aged organelles through lysosomal degradation in the cell body. Consistent with this, we provide evidence that retrograde mitochondrial transport is important for removing aged organelles from axons; however, we show that these organelles are not solely degraded, rather they persist in neurons for days. Disrupting retrograde mitochondrial transport impacts the homeostatic distribution of mitochondria throughout the neuron and the function of motor, but not sensory, axon synapses. Together, our work shows the conserved reliance on retrograde mitochondrial transport for maintaining a healthy mitochondrial pool in neurons and illustrates the disparate effects of disrupting this process on sensory versus motor circuits.

## Introduction

Mitochondria are essential organelles with vital roles in multiple cellular processes (Rizzuto et al., 1993; Verdin et al., 2010; Hall et al., 2012; Cagin et al., 2015; Kwon et al., 2016; Zheng et al., 2016; Ward and Cloonan, 2019). In neurons, mitochondria have been shown to localize to the synapse where they are critical for maintenance of local ATP levels essential for synaptic vesicle recycling, synaptic activity, and synaptic remodeling (Attwell and Laughlin, 2001; Verstreken et al., 2005; Faits et al., 2016; Smith et al., 2016). Additionally, this organelle participates in the active buffering of calcium in the axon terminal which can modulate presynaptic release (Werth and Thayer, 1994; David and Barrett, 2003; Kang et al., 2008). Therefore, maintaining a healthy pool of mitochondria is thought to be critical for the maintenance of functional neural circuits. While much is known about how mitochondria are cleared from axons after damage (Ashrafi et al., 2014; Lin et al., 2017; Zheng et al., 2019), less is known about how populations of healthy mitochondria are maintained in neuronal compartments over the long lifespan of a neuron.

Mitochondria move throughout the neuron using kinesin-1 and cytoplasmic dynein motors for anterograde and retrograde transport, respectively (Schnapp and Reese, 1989; Tanaka et al., 1998; Hollenbeck and Saxton, 2005; Pilling et al., 2006). The recruitment of motors to mitochondria is mediated by adaptor proteins. For anterograde transport, Rho GTPase 1 (Rhot1) and Trak1/2 are the most well studied adaptors (Stowers et al., 2002; Guo et al., 2005). While Trak1 binds with both kinesin-1 and dynein, Trak2 has been shown to interact preferentially with dynein, although a direct link between Trak2 function and retrograde mitochondrial transport in axons has not been explored (van Spronsen et al., 2013). Previous work from our group identified Actr10 as an important link between dynein and mitochondria. Loss of Actr10 in zebrafish neurons results in impaired retrograde mitochondrial transport but anterograde mitochondrial transport and the transport of other cargos are unaffected (Drerup et al., 2017).

In addition to the mechanics of mitochondrial movement, progress has been made in our understanding of the functional importance of mitochondrial transport in neurons. Anterograde transport of mitochondria is essential for axon outgrowth and regeneration and to deliver healthy mitochondria from the cell body to axon terminals (Morris and Hollenbeck, 1993; Ruthel and Hollenbeck, 2003; Spillane et al., 2013; Han et al., 2016; Zhou et al., 2016). To date, the primary function attributed to retrograde transport of mitochondria is the removal of damaged organelles from the distal axon, presumably to target them for degradation in the cell body. Treatment with drugs that disrupt mitochondrial physiology increases retrograde mitochondrial transport and mitophagy in the soma (Miller and Sheetz, 2004; Cai et al., 2012). Whether retrograde transport is important for maintenance of the mitochondrial population and neural circuit function in a wild-type (WT) neuron was unknown.

We used live imaging of mitochondrial population dynamics *in vivo* in zebrafish posterior lateral line (pLL) sensory and primary motor neurons to address these open questions. The pLL mechanosensory system develops within the first days of development and is functionally mature by 4 d postfertilization (dpf; Metcalfe, 1985; Kindt et al., 2012). Additionally, these axons are planar and lie just under the skin, making analyses of mitochondrial localization and dynamics straightforward (Faucherre et al., 2009; Sarrazin et al., 2010; Dow et al., 2018). Similarly, functional motor neuron axons develop within the first days of development, extending from the ventral spinal cord into the trunk (Myers, 1985; Myers et al., 1986). Using these systems, we show that mitochondria in zebrafish neurons use retrograde movement to maintain a balanced distribution of healthy mitochondria throughout the neuron. In sensory neurons, disrupting retrograde mitochondrial motility leads to accumulation of damaged organelles in axon terminals and a ∼50% loss of organelle load from the cell body. By labeling and tracking mitochondria in these axons, we demonstrate that they persist for days and can be found redistributed in all neuronal compartments in the days following labeling. Rather than complete organelle degradation by mitophagy, our results suggest that mitochondria which are retrogradely transported back to the cell body can be recycled. Disrupting this process appears to have minimal effects on postsynaptic sensory axon function, although this interpretation is complicated by the presence of other defects in this synaptic niche. Conversely, presynaptic motor neuron axon function is significantly impaired when retrograde mitochondrial transport is perturbed. Together, our data support a model in which retrograde movement supports a balanced mitochondrial distribution essential to maintaining mitochondrial health and function in neurons.

## Materials and Methods

### 

#### Zebrafish strains and husbandry

All zebrafish (*Danio rerio*) work was done in accordance with the NICHD/NINDS IACUC guidelines (protocol ID Drerup18.008 or protocol Kindt 1362-13). Adult animals were kept at 28°C and spawned according to established protocols (Westerfield, 1993). Embryos and larvae were kept in embryo media at 28°C and developmentally staged using established methods (Kimmel et al., 1995). Transgenic lines used include: *TgBAC(neurod:egfp)^nl1^* (Obholzer et al., 2008), *Tg(hsp70l:GCaMP6s-CAAX-SiLL1)^idc8Tg^* (Zhang et al., 2018), *Tg(*−*6myo6b:GCaMP6s-CAAX)^idc1Tg^* (Zhang et al., 2018), *Tg(5kbneurod:G-GECO)^nl19^* (Mandal et al., 2018), and *Tg(5kbneurod:mito-R-GECO)^nl20^* (Mandal et al., 2018). The *Tg(myo6b:mRFP-actr10)^y610,^ Tg(she:actr10p2amRFP)^y623^*, and *Tg(5kbneurod:mRFP-actr10)^nl21^* stable trangenic lines were derived using Tol2-mediated transgenesis and the Gateway system according to established protocols (Kwan et al., 2007) and previously identified promotors (Kindt et al., 2012; Quillien et al., 2017). *actr10^nl15^* and *p150b^nl16^* mutants were genotyped as previously described (Drerup et al., 2017). The *p150a* mutant was created using CRISPR-Cas9 gene editing. Cas9 protein (Integrated DNA Technologies) was co-injected with ∼200 pg of a guide RNA targeting exon 2 of *p150a* (GGTAAGATGAGTTCAGACGG). A mutant line was identified with a 23 base pair deletion in exon 1 of *p150a*. This *p150a^y625^* line recapitulates the small eye and pigment phenotypes associated with a previously identified *p150a* loss of function allele (Del Bene et al., 2008).

#### Transient transgenesis

For analyses of mitochondrial localization and measures in single neurons, we used transient transgenesis for mosaic expression. Plasmid DNA encoding the *5kbneurod* (pLL neurons; (Mo and Nicolson, 2011) or *mnx1* (motor neurons; (Flanagan-Steet et al., 2005; Palaisa and Granato, 2007) promotors driving constructs of interest were derived using Gateway technology (Kwan et al., 2007). For expression, 3–13 pg of plasmid DNA was microinjected into zebrafish zygotes as previously described (Drerup and Nechiporuk, 2013, 2016). For analysis, larvae expressing the construct of interest in a subset of pLL or motor neuron cell bodies were selected using a Zeiss AxioZoom fluorescent dissecting scope. Larvae were anesthetized in 0.02% tricaine and mounted individually in 1.5% low melt agarose in embryo media and imaged with a Zeiss LSM800 confocal microscope with a 63× (NA1.2) water immersion objective.

#### Mitochondrial photoconversion and time-lapse imaging

mEos localized to the intermembrane space of mitochondria in axon terminals (using the localization signal sequence from Cox8) was converted using a 405-nm laser on the Zeiss LSM800 confocal microscope with a 40×/NA1.0 dipping lens. For conversion, larvae were mounted in 0.8% low melt agarose and immersed in embryo media with 0.02% tricaine. A preconversion image was taken, and then a region of interest (ROI) was defined around the area to be converted. A *z*-stack with a range of 9–25 µm was set up, and the ROI was scanned with 5–7% 405-nm laser power one or two times for complete conversion. Conversion was confirmed with a postconversion image. For the 24-h time point, larvae were housed in embryo media at 28°C overnight in the dark before being imaged again the following day. For time-lapse imaging, *z*-stacks through the ROI were set up to be scanned every 1, 3, or 10 min for 1–16 h. All imaging was done using the 488 nm (1% power) and 568 nm (1% power) lasers on a LSM800 Zeiss confocal microscope with a 40×/NA1.0 dipping objective.

For whole larval conversion (Fig. 10), larvae were converted using epifluorescence and a DAPI filter on an AxioZoom V.16 Zeiss microscope. Images were taken 6 and 24 h postconversion (hpc) on an LSM800 confocal microscope with a 40×/NA1.0 dipping objective but with 20% 568-nm laser power to visualize trace amounts of converted mEos.

For the mEos tracking experiments in Figs. 7–9, all or a small region of mitochondria in an axon terminal was converted and imaged using the LSM800 confocal microscope with a 40×/NA1.0 dipping objective; 24, 48, and 72 hpc time points are independent experiments. Imaging was done using the 488 nm (1% laser power) and 568 nm (20% laser power).

#### Fluorescence intensity from mosaic expression and mitochondrial area analyses

Within experiments quantifying fluorescence intensity, all settings including laser power, gain, *z*-step size, and image resolution were kept consistent between all samples. The neuronal area analyzed was defined either manually by tracing or by masking using a cytoplasmic fill and the ImageJ image calculator plugin. Mean fluorescence intensity was then calculated from a sum projected *z*-stack using ImageJ (Schindelin et al., 2012).

For quantification of areas, the cell body or axon terminal to be analyzed was defined by manual tracing or by masking using a GFP neuronal cell fill in a projected *z*-stack. For quantification of mitochondrial area, a threshold was manually applied to a projected SD *z*-stack in ImageJ and the area masked selected and quantified using the measure plugin.

#### Analysis of synaptic vesicle release using SypHy

SypHy and cytoplasmic mRFP were co-expressed in motor neurons using mosaic expression from DNA injection into zygotes. SypHy and mRFP were both driven by the *mnx1* promotor as described above using a p2a sequence to separate the proteins. This allows normalization to mRFP as both should be expressed at equal levels. For SypHy analysis, larvae were sorted for mRFP expression in the ventral spinal cord on a Zeiss AxioZoom fluorescent stereoscope. At 4 dpf, these larvae were incubated in tricaine (0.02%) with or without NMDA (100 μm) for 2 min. After incubation, larvae were washed in fresh embryo media and mounted as described above before being imaged on a Zeiss LSM800 confocal microscope with a 63×/NA1/2 water immersion objective. After identifying a motor neuron axon terminal in the trunk, a *z*-stack of the region was taken every 10 s for 5 min. Imaging settings were kept consistent between animals. For analysis, SypHy and mRFP mean fluorescence intensities were measured in sum projected *z*-stacks of the axon terminal at each time point using ImageJ. Cytoplasmic mRFP served as an internal control and normalization factor for expression level. Statistical analysis was performed on average SypHy mean fluorescence intensity normalized to mRFP mean fluorescence intensity.

#### Immunohistochemistry and vital dye labeling

For hair cell (HC) synapse immunolabeling, zebrafish larvae at 5 dpf were fixed in 4% PFA in PBS for 3.5 h at 4°C. After rinse, larvae were then permeabilized in acetone (stored at −20°C) for 5 min and blocked with PBST buffer containing 2% goat serum, 2% fish skin gelatin and 1% BSA, overnight at 4°C. Primary antibodies were diluted in block solution. After removal of primary antibody, larvae were incubated in Alexa Fluor-conjugated secondary antibodies. Larvae were then mounted on slides with ProLong Gold Antifade Reagent (Life Technologies). Fixed samples from HC synapse immunolabeling were imaged on an inverted Zeiss LSM 780 laser-scanning confocal microscope using a 63×/1.4 NA oil objective lens. Images were acquired with a 5.0 × zoom at 620 × 620 every 0.19 μm. The *z*-stacks were processed with Zeiss Zen Black software v2.1 using an Airyscan processing factor optimized with auto feature setting: Ribeye (7.5–7.8) and MAGUK (6.0–6.3). Synapses and HCs were counted manually. Counts were done blinded. Myosin VIIa label was used to determine HC number.

To quantify HC presynapse and postsynapse size, images were processed in ImageJ. In ImageJ, each Airyscan *z*-stack was background subtracted using rolling-ball subtraction. *z*-stacks containing the MAGUK channel were further bandpass filtered to remove details smaller than 6 px and larger than 20 px. A duplicate of the *z*-stack was normalized for intensity. This duplicated *z*-stack was used to identify individual ribbon and MAGUK using the Simple 3D Segmentation of ImageJ 3D Suite (Ollion et al., 2013). Local intensity maxima, identified with 3D Fast Filter, and 3D watershed were used to separate close-by structures. The max *z*-projection of the segmented *z*-stack was used to generate a list of 2D objects as individual ROIs corresponding to each punctum. This step also included a minimum size filter, Ribeye: 0.08 µm^2^, MAGUK 0.04 µm^2^. Area and intensity measurements from these ROIs were exported from ImageJ.

For neuromuscular junction labeling, 4 dpf larvae were fixed in 4% paraformaldehyde/0.25% triton and washed in water overnight at room temperature (RT). They were then blocked in AB block (0.1% Triton, 1% dimethylsulfoxide (DMSO), 0.02% Sodium azide, 0.5% BSA, and 5% goat serum) overnight before incubation in anti-SV2 (1:100) overnight in AB block. Larvae were then washed in PBS-0.1% Triton X-100 before incubation in anti-mouse Alexa Fluor 568 (1:1000) and α-bungarotoxin-647 (10 μm; ThermoFisher Scientific) overnight. After washing in PBS-0.1% Triton X-100, larvae were sunk and imaged in 60% glycerol in PBS-0.1% Triton X-100. Images were taken on a LSM800 Zeiss confocal microscope with a 20×/NA0.8 objective. For quantification of presynapse and postsynapse area, two dorsal segments from each animal were isolated in ImageJ from a SD projected *z*-stack. A mask was made using the ImageJ Threshold tool with default settings and the area selected by thresholding and measured using built-in plugins.

Vital dye labeling with tetramethylrhodamine ethyl ester (TMRE) was performed as previously described (Mandal et al., 2018). Briefly, zebrafish larvae at 4 dpf were incubated in 25 μm TMRE in embryo media with 0.1% DMSO for 1 h in the dark. Larvae were subsequently washed three times in embryo media before being anesthetized in 0.02% tricaine in embryo media, mounted in 1.5% low melt agarose, and imaged with a 63×/NA1.4 objective on a confocal microscope (Zeiss LSM800). These experiments were done in carefully timed batches so larvae were in TMRE for precisely 1 h to prevent signal decay before imaging. For analysis, mitochondrial TMRE mean fluorescence intensity was measured in sum projections of axon terminals after subtraction of nonneural tissue using the ImageJ *Image Calculator* function using the GFP fill from the *TgBAC(neurod:egfp)^nl1^* transgenic line.

#### Hair-cell stimulation and pLL functional imaging

Larvae at 4–6 dpf were prepared for GCaMP6s calcium imaging as described previously (Lukasz and Kindt, 2018). Primary posterior lateral-line neuromasts (NM1-NM4) were stimulated using a fluid jet. The fluid jet consisted of a pressure clamp (HSPC-1, ALA Scientific) attached to a glass pipette (inner tip diameter, ∼50 μm). The glass pipette was filled with solution and used to deliver a 500-ms anterior and posterior mechanical stimulus. For GCaMP6s Ca^2+^ imaging a Bruker Swept-field confocal system with Prairie View software (Bruker Corporation) was used for image acquisition. The system was equipped with a Rolera EM-C2 CCD camera (QImaging) and a Nikon CFI Fluor 60×/NA 1.0 water immersion objective. For calcium imaging, five plane *z*-stacks were acquired every 0.5 µm (HC Apex calcium) or 1 µm (HC Base, presynaptic or postsynaptic calcium), at a 50-Hz frame rate, yielding a 10-Hz volume rate. *Z*-stacks were acquired using a piezoelectric motor (PICMA P-882.11-888.11 series, PI Instruments) attached to the objective to allow rapid imaging along the *z*-axis. Five plane *z*-stacks were projected into a single plane for further image processing and quantification. After postsynaptic GCaMP6s calcium measurements, larvae were labeled and with FM 4–64 (3 μm for 30 s) to label HCs.

After imaging, the raw images were registered to reduce movement artifacts. For presynaptic and postsynaptic GCaMP6s measurements, a circular ROI with a diameter ∼3 µm was placed on the afferent process beneath an active HC within a neuromast. After selecting an ROI, we calculated and plotted (ΔF/F_0_) within each ROI during the recording period. The signal magnitude was defined as the peak value of intensity change on stimulation. Spatial heat maps to display calcium signals were generated as described previously (Zhang et al., 2016; Lukasz and Kindt, 2018). Heatmaps from anterior and posterior stimuli were combined into a single image for simplicity.

#### Motor neuron calcium imaging

Larvae at 4–5 dpf were immobilized in a droplet of 1% low melt agarose on a Sylgard-lined plate. To suppress larvae movement, α-bungarotoxin was injected into the heart. Agarose surrounding the tail was then removed. Before imaging, larvae in low melt agarose were submerged in embryo media.

To measure presynaptic calcium activities in motor neuron presynapses we used a cell-fill G-Geco in the *Tg(5kbneurod:G-GECO)^nl19^* transgenic line. For calcium imaging, we used the Bruker Swept-field confocal system described for pLL functional imaging. G-Geco was excited using a 488-nm laser and emission was captured using a dual bandpass 488/561-nm filter set (59904-ET, Chroma). G-Geco images were acquired in a single plane at a 20-Hz frame rate. During image acquisition, the exposed tail was deflected using a fluid-jet delivered by a glass pipette (inner tip diameter, >35 µm) filled with embryo media. Stimulation was delivered 5 s after imaging began. Positive pressure was delivered by the fluid jet on the larvae tail for 50 ms with minimum of 2-min rest in between stimulations.

To quantify G-Geco intensity changes from the calcium imaging, raw images were processed using FIJI (Schindelin et al., 2012). Images were registered in X-Y to reduce movement artifacts using the Image Stabilizer plugin (Li, 2008). To segment the frame for intensity measurement, we used Plot Profile at frame 60, or 3 s after imaging began, to plot the intensity value across an arbitrary axis of the visible motor neuron. The resulting intensity plot of frame 60 was fitted to a Gaussian curve to estimate the mid-point between baseline and maximum intensity. This half-max value was used as the minimum threshold value to segment the image into regions, from which the region encompassing the motor neuron was selected to measure the fluorescent intensity over the entire movie. The average ROI generated encompassed 4.2 ± 0.98 µm^2^ for WT fish and 6.0 ± 1.7 µm^2^ for *actr10^nl15^* mutants. The average intensity for 2 s preceding stimulation was used as baseline to calculate the normalized intensity (ΔF/F) across the temporal trace.

#### Behavioral analysis

For spontaneous swim recording, a Zantiks MWP behavioral measurement system was used to track larvae between 5 and 10 h after A.M. light onset. A 12-well plate was mounted on an acrylic platform in this system. Each well was filled with embryo media or 100 μm NMDA in embryo media (5–6 ml). Larvae were acclimated to darkness for 15 min in embryo media before each recording session (Bhandiwad et al., 2018). During the measurements, larvae were illuminated with an infrared light source from below and recorded from the top using a built-in infrared camera at 30 frames per second with 48.2 pixels/centimeter resolution. Each larva was recorded for a single 20-min session, and the first 5 min were used for analysis.

Video recorded in the Zantiks equipment was relayed to a connected Cisco router for processing and temporary storage. From the video, distance traveled was derived by the built-in tracking algorithm from the change in *x*, *y* centroid coordinates collected every 0.033 s and binned every 0.3 s. Tracking data were manually checked for false positives or negatives by comparing the distance-traveled values against video recordings. Similar number of WT and mutant fish (identified by phenotype) were measured in each clutch in each experimental day to account for environmental and clutch-to-clutch variabilities. In *actr10* neuronal rescue recordings, mutants cannot be phenotypically distinguished from WT and were randomly distributed across experimental days. All fish were genotyped after recording.

#### Lysosomal inhibition

Inhibition of lysosomal degradation was done using a previously published combination of pharmacological treatments (Tanida et al., 2005; He et al., 2009). For this experiment, larvae were immobilized and mitochondria in axon terminals were photoconverted as described above. Immediately after photoconversion, larvae were removed from the agarose and placed in 10µM Pepstatin A (Fisher Scientific; BP26715)/10µM E64D (Enzo Life Sciences; BML-PI107-0001) in embryo media with 0.1% DMSO overnight before being imaged again the following day as described above.

#### Cortical neuron culture analysis

Primary hippocampal neurons were isolated from embryonic day (E)17–E18 rat embryos as described previously (Roche and Huganir, 1995). Neurons were co-transfected with 500 ng each of EGFP and pSuper vector or Actr10 shRNA #2 (ggtcctggattagtggatatag) at 13 days in vitro (DIV) using Lipofectamine 2000; 4 d posttransfection, at DIV17, the cells were fixed and stained for endogenous TOM20, a mitochondrial marker. Fixation was performed in PBS containing 4%PFA and 4% sucrose for 7–8 min, followed by permeabilization with 0.25% Triton X-100 in PBS for 10 min. TOM20 staining was done using anti-Tom20 for 1 h at RT, followed by staining with anti-rabbit Alexa Fluor 555 (1:1000, Life Technologies) for 1 h at RT. The slides were mounted in Prolong gold antifade and imaged using a Zeiss LSM800 confocal microscope with a 63×/NA 1.4 objective. Cell somas in 7–21 neurons were analyzed per coverslip. Five slides with two coverslips each were analyzed per condition in two blinded, experimental replicates.

#### Statistical analyses

For analyses of synapse number and size, sensory afferent axon activity, motor neuron activity and behavioral assays, statistical analysis was performed using Prism 8 (GraphPad). For analyses of synapse number and size statistical significance was determined by *t* test with a Dunnett's test to correct for multiple comparisons (synapse counts) or an unpaired *t* test (areas). For analyses of sensory afferent axon activity, statistical significance was determined by one-way ANOVA with a Dunnett's test to correct for multiple comparisons. For motor neuron activity measurements statistical significance was determined by Kruskal–Wallis and Dunn's *post hoc* or Mann–Whitney *U* test as appropriate. All calcium imaging experiments were performed on a minimum of three animals and on three independent days. Correlational analyses were done using the Coloc2 feature in ImageJ and the Pearson correlation coefficient is reported.

For all other experiments, all statistical analyses were done using JMP14. For parametric analyses, ANOVAs were used with Tukey's HSD *post hoc* contrasts for pairwise comparisons. For non-parametric datasets, comparisons were done using Wilcoxon/Kruskal–Wallis analyses with corrected tests for each pair for multiple comparisons. Outliers were defined as data points that were outside of 3 SDs from the mean.

Data in text or plotted with error bars on graphs were expressed as mean ± SEM.

## Results

### Inhibition of retrograde mitochondrial transport leads to an imbalance in neuronal mitochondrial load

**Figure 1.**
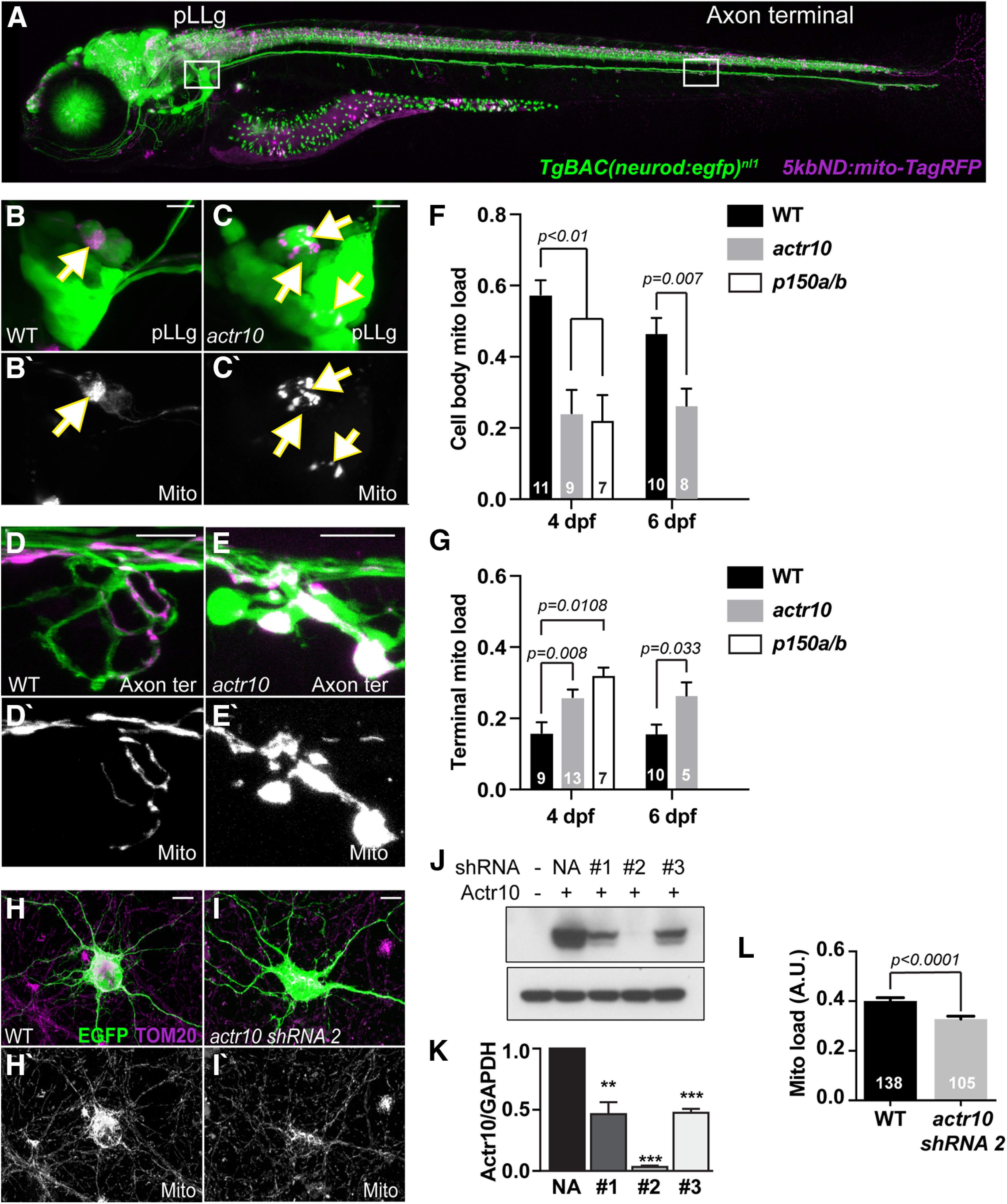
Disruption of retrograde mitochondrial movement impacts mitochondrial distribution in neurons. ***A***, Image of a 4 dpf *TgBAC(neurod:egfp)^nl1^* transgenic zebrafish larva expressing mitochondrially localized TagRFP (shown in magenta) mosaically in neurons. Regions imaged in ***B***, ***D*** shown in white boxes. ***B***, ***C***, pLLg neurons have cytosolic GFP and a subset express TagRFP in mitochondria (arrows). In WT animals, mitochondria (magenta in ***B***; white in ***B'***) fill the soma. ***C***, In *actr10^nl15^* mutants, cell body mitochondrial area is reduced. ***D***, ***E***, Mitochondria accumulate in axon terminals of *actr10^nl15^* mutants. ***F***, Quantification of cell body mitochondrial load (mitochondrial area/cell body area) at 4 dpf shows a reduction in *actr10^nl15^* and *p150a/b* mutants (ANOVA); 4 dpf, WT: 0.58 ± 0.04; *actr10^nl15^*: 0.24 ± 0.04; *p150a/b*: 0.21 ± 0.05; 6 dpf, WT: 0.47 ± 0.04; *actr10^nl15^*: 0.26 ± 0.05. ***G***, Axon terminal mitochondrial load (mitochondrial area/axon terminal area) at 4 dpf is increased in *actr10^nl15^* and *p150a/b* mutants (ANOVA). Changes in mitochondrial load are also observed at 6 dpf in *actr10^nl15^* animals; 4 dpf – WT: 0.16 ± 0.03; *actr10^nl15^*: 0.26 ± 0.03; *p150a/b*: 0.31 ± 0.03; 6 dpf, WT: 0.16 ± 0.03; *actr10^nl15^*: 0.26 ± 0.04. ***H–N***, shRNA-mediated knock-down of Actr10 in rat hippocampal neurons results in loss of cell body mitochondrial load. Images of hippocampal neuron cell bodies (***H***, ***I***) stained for TOM20 with a cytoplasmic EGFP fill and either vector only (***H***) or *actr10* shRNA#2 co-transfection (***I***). Arrows points to mitochondrial in axon terminals. ***J***, ***K***, Western blotting and quantification showing knock-down of Actr10 by the shRNAs tested in HEK cells (top, Actr10; bottom, GAPDH; **p < 0.01, ***p < 0.005; ANOVA; *n* = 3). ***L***, Quantification of mitochondrial load in Actr10 knock-down hippocampal neurons (ANOVA). WT: 0.40 ± 0.01; *actr10* shRNA2: 0.32 ± 0.01. Sample sizes (***F***, ***G***, number of larvae; ***L***, number of neurons) indicated on graph. Scale bars: 10 µm. All data are mean ± SEM.

Retrograde mitochondrial transport has previously been linked to degradation of unhealthy organelles. We were curious about the role of retrograde mitochondrial movement in maintenance of the mitochondrial population in toto in neurons. To begin to investigate this, we analyzed the distribution and health of mitochondria in a mutant zebrafish line in which retrograde mitochondrial transport is inhibited. Previously, we characterized a novel zebrafish line, *actr10^nl15^*, in which retrograde mitochondrial transport in neurons is disrupted. In this line, loss of Actr10 leads to inhibition of retrograde mitochondrial movement with no change in the transport or localization of other cargos assayed. Additionally, anterograde mitochondrial transport is intact (Drerup et al., 2017). To determine whether overall mitochondrial distribution was altered with this specific disruption in retrograde mitochondrial transport, we used a sparse labeling approach. We injected a *5kbneurod:mito-TagRFP* DNA plasmid into zygotes for mosaic expression of mitochondrially localized TagRFP in a subset of pLL sensory neurons (Drerup and Nechiporuk, 2016; Mandal et al., 2018). We used this method to image mitochondrial populations in the cell body and axon terminals of pLL sensory neurons with and without retrograde mitochondrial transport disruption (Fig. 1*A*). We performed this analysis at 4 dpf, after synaptic contacts between afferent axons and sensory organs are established (Sheets et al., 2012), and at 6 dpf, when synapses are mature and myelination of this system has been initiated (Brösamle and Halpern, 2002; Monk et al., 2009). We observed that mitochondria fill the cell body of WT neurons at 4 dpf and are distributed throughout the axon and axon terminal. In contrast, inhibition of retrograde mitochondrial movement in the *actr10^nl15^* mutant line leads to a significant reduction of mitochondrial load in the soma and an accumulation of this organelle in axon terminals (soma: *F*_(1,20)_ = 9.006, *p* = 0.0071, ANOVA; axon terminal, *F*_(1,21)_ = 8.683, *p* = 0.0077, ANOVA; Fig. 1*B–G*). This abnormal mitochondrial distribution persists at 6 dpf (soma: *F*_(1,16)_ = 9.563, *p* = 0.0070, ANOVA; axon terminal, *F*_(1,13)_ = 5.688, *p* = 0.0330, ANOVA; Fig. 1*F*,*G*). To confirm that loss of retrograde mitochondrial movement contributed to this defect, we also looked at mitochondrial distribution in zebrafish *p150a/b* mutants. In this mutant line, the essential dynein cofactor dynactin is lost, leading to a loss of all retrograde cargo transport, including that of mitochondria (Drerup et al., 2017). On the background of a preexisting *p150b* zebrafish mutant (Drerup et al., 2017), we used CRISPR-Cas9 mediated gene editing to create a *p150a/b* double mutant with a 23-bp deletion in exon 1 of the *p150a* paralog. This mutant perfectly phenocopies a previously characterized *p150a* mutant (Del Bene et al., 2008). Analysis of mitochondrial localization in this line revealed that similar to loss of Actr10, loss of p150 caused a shift in the distribution of mitochondria toward the axon terminal at 4 dpf (soma: *F*_(1,18)_ = 10.607, *p* = 0.0044, ANOVA; axon terminal, *F*_(1,15)_ = 8.467, *p* = 0.0108, ANOVA; Fig. 1*F*,*G*).

Finally, we asked whether this mitochondrial distribution phenotype was zebrafish specific or whether it could be found in a mammalian model system. To address this, we used siRNA-mediated knock-down of Actr10 in cultured rat hippocampal neurons and found that this significantly reduced cell body mitochondrial load similar to what we observed in *actr10^nl15^* mutants (*F*_(1,241)_ = 22.513, *p* < 0.0001, ANOVA; Fig. 1*H–L*). Together, our data from zebrafish neurons and hippocampal neurons supports a role for active retrograde mitochondrial transport in the distribution of mitochondria throughout the neuron.

### Failed mitochondrial health but normal ATP levels in *actr10^nl15^* pLL axon terminals

**Figure 2.**
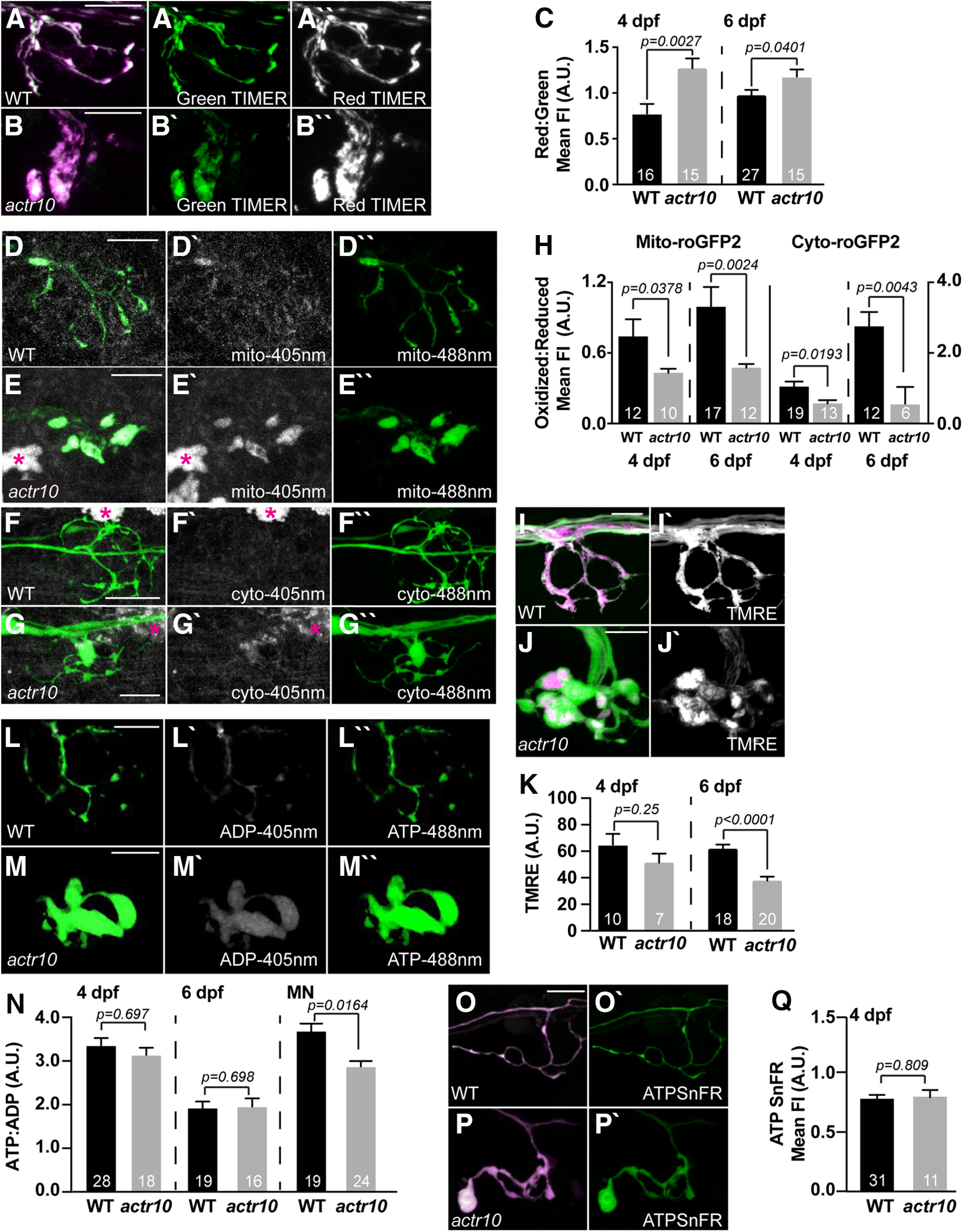
Loss of retrograde mitochondrial transport results in altered measures of mitochondrial health. ***A***, ***B***, TIMER fluorescence in the intermembrane space of mitochondria in the axon terminal of WT and *actr10^nl15^* mutants at 4 dpf. Oxidized TIMER, magenta/white; reduced TIMER, green. ***C***, Quantification of the oxidized:reduced TIMER protein at 4 and 6 dpf demonstrates cumulative oxidation of the TIMER protein in axon terminal mitochondria with retrograde transport reduction (ANOVA); 4 dpf, WT: 0.78 ± 0.10; *actr10^nl15^*: 1.27 ± 0.11; 6 dpf, WT: 0.98 ± 0.06; *actr10^nl15^*: 1.18 ± 0.08. ***D–H***, roGFP2 reveals acute changes in ROS levels in mitochondria of *actr10^nl15^* mutants. ***D***, ***E***, Mitochondrially localized roGFP2 (localized to the intermembrane space) in axon terminals of WT and *actr10^nl15^* mutants at 4 dpf. ***F***, ***G***, Cytoplasmic roGFP2 expression in axon terminals of WT and *actr10^nl15^* mutants at 4 dpf. Asterisks on autofluorescent pigment cells. ***H***, Quantification of the ratio of oxidized to reduced roGFP2 in mitochondria (left) or cytosol (right) of the axon terminal at 4 and 6 dpf (ANOVA or Wilcoxon rank-sum). Mitochondrial: 4 dpf, WT: 0.74 ± 0.11; *actr10^nl15^*: 0.43 ± 0.12; 6 dpf, WT: 0.99 ± 0.13; *actr10^nl15^*: 0.47 ± 0.16. Cytoplasmic: 4 dpf, WT: 1.08 ± 0.13; *actr10^nl15^*: 0.56 ± 0.16; 6 dpf, WT: 2.88 ± 0.45; *actr10^nl15^*: 0.66 ± 0.64. ***I***, ***J***, TMRE staining (magenta in merge; white in single channel) of the mitochondrial matrix in axon terminals at 4 dpf. Neurons are labeled with cytosolic GFP. ***K***, Mean TMRE fluorescence is slightly reduced at 4 dpf and significantly decreased in axon terminal mitochondria at 6 dpf when retrograde mitochondrial transport is inhibited (ANOVA); 4 dpf, WT: 64.98 ± 8.07; *actr10^nl15^*: 52.41 ± 6.75; 6 dpf, WT: 61.94 ± 3.08; *actr10^nl15^*: 37.89 ± 2.92. ***L***, ***M***, WT and *actr10^nl15^* mutant axon terminals expressing PercevalHR at 4 dpf. ***N***, Quantification of the ATP:ADP ratio at 4 and 6 dpf shows no change in *actr10^nl15^* mutants (ANOVA); 4 dpf, WT: 3.40 ± 0.20; *actr10^nl15^*: 3.09 ± 0.25; 6 dpf, WT: 1.81 ± 0.20; *actr10^nl15^*: 1.70 ± 0.20. ATP:ADP ratios are decreased in motor neuron (MN) axons at 4 dpf in *actr10^nl15^* mutants (ANOVA). WT: 3.73 ± 0.20; *actr10^nl15^*: 2.78 ± 0.18. ***O–Q***, ATPSnFR analysis of cytosolic ATP levels at 4 dpf. Magenta, cytosolic mRuby; green, ATPSnFR. ***O***, ***P***, Expression of *5kbneurod:mRuby-ATPSnFR* in a single WT and *actr10^nl15^* mutant axon terminal. ***Q***, Quantification of the ATPSnFR fluorescence intensity normalized to mRuby expression shows no difference in ATP levels between WT and *actr10^nl15^* mutant axon terminals (ANOVA). WT: 0.80 ± 0.03; *actr10^nl15^*: 0.81 ± 0.06. Sample sizes indicated on graph. Scale bar: 10 µm. All data are mean ± SEM.

Mitochondrial retrograde transport is thought to clear aged or damaged organelles from the axon. To determine whether inhibition of retrograde transport impacts mitochondrial health in axon terminals, we used various fluorescence indicators in *actr10^nl15^* mutants. One indicator of health is accumulation of reactive oxygen species (ROS). While ROS production is a normal part of ATP synthesis, chronic accumulation of damaging levels of this metabolic by-product correlate with failed mitochondrial and neuronal health (Letai, 2006; Schon and Przedborski, 2011; Williams et al., 2013; Andreyev et al., 2015). First, we analyzed chronic ROS exposure using the fluorescent ROS sensor TIMER localized to the mitochondrial intermembrane space (Fang et al., 2012). TIMER is a fluorescent protein that fluoresces in the green (488 nm) spectrum in its native state but irreversibly switches to red (568 nm) on oxidation (Terskikh et al., 2000; Hernandez et al., 2013; Laker et al., 2014). This indicator has been used previously to assay chronic exposure to ROS in zebrafish (Mandal et al., 2018; Pickett et al., 2018). We used mosaic expression of mitochondrially localized TIMER in individual pLL neurons and assayed the red:green fluorescence ratio at 4 and 6 dpf in *actr10^nl15^* mutants and WT siblings. This analysis revealed a consistent increase in the red:green TIMER ratio in axon terminal mitochondria when retrograde transport is inhibited, indicating that these organelles chronically accumulate more ROS (4 dpf, *F*_(1,29)_ = 10.742, *p* = 0.0027, ANOVA; 6 dpf, *F*_(1,40)_ = 4.500, *p* = 0.0401, ANOVA; Fig. 2*A–C*). It is important to note that TIMER measures are confounded by mitochondrial age, which does not differentiate between (1) a greater rate of ROS production and (2) longer-term exposure to ROS.

To clarify these findings, we assayed acute ROS in the axon terminal mitochondrial population of *actr10^nl15^* mutants. For this, we used a reversible ROS indicator, roGFP2 (Dooley et al., 2004; Hanson et al., 2004). roGFP2 has peak fluorescence in the 405 nm spectrum when oxidized and 488 nm spectrum when reduced. Because it is reversible, this protein gives a real time read-out of oxidative state of roGFP2. We used mosaic expression of either cytosolic (cyto-) or mitochondrially localized (mito-) roGFP2 in pLL neurons and assayed the oxidized to reduced roGFP2 ratio in axon terminals (Fig. 2*D–G*). At both 4 and 6 dpf, we observed similar trends: mitochondria in retrograde transport mutants show a lower oxidized:reduced mito-roGFP2 fluorescence ratio in axon terminals compared with WT siblings. This was mirrored by cyto-roGFP2 fluorescence ratios [mito-roGFP2: 4 dpf (*Z* = −2.077, *p* = 0.0378, Wilcoxon); 6 dpf (*Z* = −3.033, *p* = 0.0024, Wilcoxon); cyto-roGFP2: 4 dpf (*Z* = −2.340, *p* = 0.0193, Wilcoxon); 6 dpf (*Z* = −2.857, *p* = 0.0043, Wilcoxon); Fig. 2*H*]. Together, our data show that when mitochondria are retained in axon terminals, they produce lower levels of ROS acutely. Therefore, the elevated measure of chronic ROS (TIMER) is likely a consequence of organelle age.

Lowered acute ROS production could indicate failing mitochondrial health. To assay mitochondrial health, we analyzed mitochondrial matrix potential in pLL axon terminals using the vital dye TMRE. This cationic dye accumulates in the negatively charged mitochondrial matrix to a degree relative to potential (Mitra and Lippincott-Schwartz, 2010; Esterberg et al., 2014). By 6 dpf, mitochondrial matrix potential was significantly reduced in the *actr10^nl15^* mutant, a sign that these aged organelles fail to maintain their matrix potential when they are retained in the axon terminal (4 dpf, *F*_(1,15)_ = 1.427, *p* = 0.251, ANOVA; 6 dpf, *F*_(1,36)_ = 32.131, *p* < 0.0001, ANOVA; Fig. 2*I–K*). Both decreased acute ROS production and matrix potential is predicted to reflect impaired oxidative phosphorylation and ATP production. To assay ATP production directly, we expressed the ratiometric sensor PercevalHR in pLL neurons and assayed the ATP (488 nm) to ADP (405 nm) fluorescence ratios in the axon terminal (Tantama et al., 2013; Mandal et al., 2018). Somewhat surprisingly, in *actr10^nl15^* mutants we observed no change in the cytosolic ATP:ADP ratio in pLL axon terminals at 4 or 6 dpf (4 dpf, *F*_(1,39)_ = 0.1544, *p* = 0.697, ANOVA; 6 dpf, *F*_(1,38)_ = 0.1525, *p* = 0.698, ANOVA; Fig. 2*L–N*). To confirm our Perceval HR results, we also assessed ATP levels at 4 dpf using a non-ratiometric sensor, ATP SnFR (Lobas et al., 2019). As we observed with PercevalHR, there was no change in the overall level of ATP in axon terminals of pLL sensory neurons despite mitochondrial dysfunction (*F*_(1,40)_ = 0.0592, *p* = 0.809, ANOVA; Fig. 2*O–Q*). These analyses demonstrated that despite deficits in mitochondrial health in sensory axon terminals, ATP levels were normal.

### Cytoplasmic ATP is reduced in *actr10^nl15^* motor neuron axons

The normal ATP level in *actr10^nl15^* pLL axon terminals was surprising as mitochondrial dysfunction is predicted to impact ATP production. However, the majority of studies on axonal ATP have been done on presynaptic axons. Sensory axon terminals, like those of the pLL, are postsynaptic, potentially changing their reliance on ATP as they do not require ATP-dependent synaptic vesicle cycling/recruitment. Vesicle recruitment in particular has been shown to rely heavily on synaptic mitochondria at the neuromuscular junction, particularly with repeated activity (Verstreken et al., 2005). To determine whether presynaptic axon ATP levels were altered, we analyzed motor neuron axon cytosolic ATP:ADP ratios in WT and *actr10^nl15^* mutants. This analysis demonstrated that, unlike sensory axons, motor neuron axons have a significant drop in ATP levels when mitochondrial retrograde transport is disrupted (4 dpf, *Z* = 2.400, *p* = 0.0164, Wilcoxon; Fig. 2*N*). This is in line with previous work which has shown that presynaptic axons rely on axonal mitochondria for maintenance of ATP levels (Rangaraju et al., 2014).

### Mitochondrial health in pLL neuronal cell bodies is intact in *actr10^nl15^* mutants

**Figure 3.**
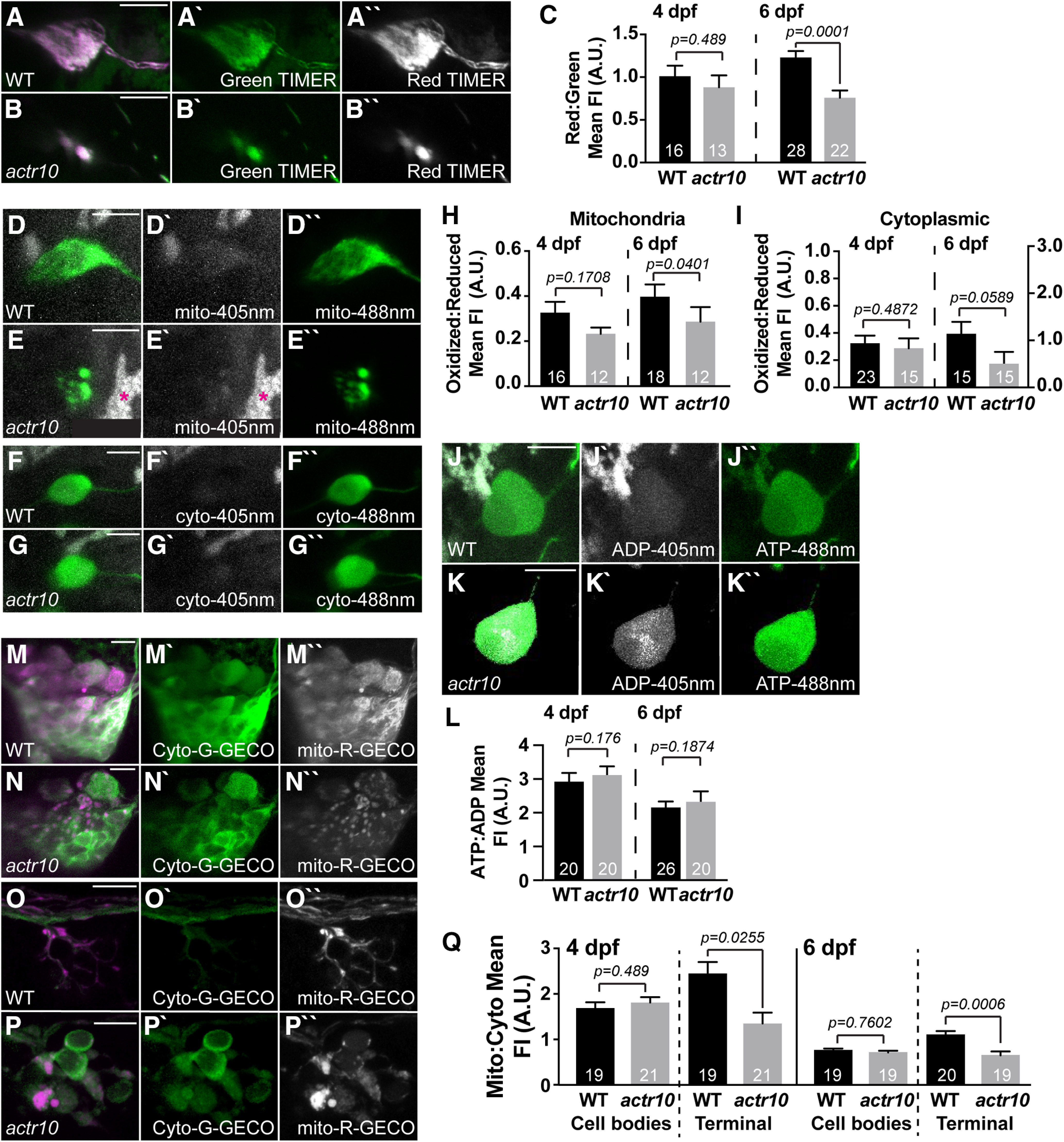
Mitochondrial calcium load is decreased in axon terminal mitochondria in *actr10^nl15^* mutants. ***A–C***, TIMER fluorescence in the cell body mitochondria show lower cumulative ROS exposure by 6 dpf with retrograde mitochondrial transport inhibition (ANOVA); 4 dpf, WT: 1.01 ± 0.12; *actr10^nl15^*: 0.88 ± 0.14; 6 dpf, WT: 1.23 ± 0.08; *actr10^nl15^*: 0.76 ± 0.08. ***D–I***, roGFP2 expression in mitochondria and the cytosol of the cell body shows no change in acute ROS at 4 or 6 dpf in *actr10^nl15^* mutants (ANOVA). Mitochondrial: 4 dpf, WT: 0.33 ± 0.04; *actr10^nl15^*: 0.23 ± 0.05; 6 dpf, WT: 0.40 ± 0.05; *actr10^nl15^*: 0.29 ± 0.07. Cytoplasmic: 4 dpf, WT: 0.34 ± 0.06; *actr10^nl15^*: 0.29 ± 0.07; 6 dpf, WT: 1.17 ± 0.27; *actr10^nl15^*: 0.52 ± 0.29. ***J–L***, Cell body ATP:ADP ratios, measured using PercevalHR expression, are unchanged in *actr10^nl15^* mutants (ANOVA); 4 dpf, WT: 3.01 ± 0.29; *actr10^nl15^*: 3.14 ± 0.29; 6 dpf, WT: 2.12 ± 0.23; *actr10^nl15^*: 2.34 ± 0.26. ***M–Q***, Cell bodies (***M***, ***N***) and axon terminals (***O***, ***P***) expressing mitochondrially localized R-GECO (magenta in merge, white in single channel) and cytoplasmic G-GECO (green) in WT and *actr10^nl15^* mutants at 4 dpf. ***Q***, Quantification of mitochondrial:cytoplasmic GECO signal at 4 and 6 dpf in the pLLg and axon terminals (ANOVA or Wilcoxon rank-sum). Ganglion: 4 dpf, WT: 1.69 ± 0.13; *actr10^nl15^*: 1.81 ± 0.12; 6 dpf, WT: 0.70 ± 0.04; *actr10^nl15^*: 0.66 ± 0.04. Axon terminal: 4 dpf, WT: 2.45 ± 0.25; *actr10^nl15^*: 1.35 ± 0.24; 6 dpf, WT: 1.02 ± 0.08; *actr10^nl15^*: 0.60 ± 0.08. Sample sizes indicated on graph. Scale bar: 10 µm. All data are mean ± SEM.

Our analyses of postsynaptic axon terminal mitochondria in pLL neurons revealed an accumulation of damaged organelles when retrograde transport is inhibited. We next addressed the health and function of mitochondria in the neuronal cell bodies of the pLL in *actr10^nl15^* mutants using the same indicators. This region shows a significant reduction in mitochondrial load in *actr10^nl15^* mutants (see Fig. 1). In contrast to pLL axon terminal mitochondria, TIMER imaging revealed decreased red:green TIMER by 6 dpf, indicating cell body mitochondria in *actr10^nl15^* mutants have decreased exposure to ROS compared with siblings (4 dpf, *F*_(1,27)_ = 0.4912, *p* = 0.489, ANOVA; 6 dpf, *F*_(1,49)_ = 17.499, *p* = 0.0001, ANOVA; Fig. 3*A–C*). Analyses of roGFP2 in the cell bodies show no significant change in acute ROS at 4 dpf, although both mitochondrial and cytoplasmic acute ROS are slightly reduced by 6 dpf (mito-roGFP2: 4 dpf, *Z* = −1.370, *p* = 0.1708, Wilcoxon; 6 dpf, *Z* = −2.053, *p* = 0.0401, Wilcoxon; cyto-roGFP2: 4 dpf, *Z* = −0.695, *p* = 0.4872, Wilcoxon; 6 dpf, *Z* = −1.889, *p* = 0.0589, Wilcoxon; Fig. 3*D–I*). Together, the TIMER and roGFP2 data indicate that mitochondria in the cell bodies have lower levels of ROS exposure in *actr10^nl15^*. This fits with the model that the bulk of mitochondrial biogenesis occurs in the cell body, making these residual organelles in the *actr10^nl15^* mutants likely younger than those in the axon. The next question we asked was whether the mitochondria residing in the cell bodies were functional. To assay this, we looked at ATP production. PercevalHR expression in single pLL neurons was used to assay the ATP:ADP ratio and shows no significant change in the *actr10^nl15^* mutants (4 dpf, *F*_(1,33)_ = 1.9134, *p* = 0.176, ANOVA; 6 dpf, *F*_(1,39)_ = 1.8002, *p* = 0.1874, ANOVA; Fig. 3*J–L*).

Overall, our analyses of ROS and mitochondrial health show a consistent deficit in mitochondrial health in the axon terminal with loss of retrograde mitochondrial transport. Cell body mitochondria, although reduced in area, show lower chronic ROS exposure, potentially because they are younger than organelles in the axon. These data support a role for retrograde transport in the localization of a healthy mitochondrial population in the axon.

### Disrupted mitochondrial health does not disrupt sensory axon terminal activity

In addition to ATP production, mitochondria have many other roles in the neuron. One activity that is intimately related to neural circuit function is calcium buffering (Werth and Thayer, 1994; David and Barrett, 2003; Verstreken et al., 2005; Kwon et al., 2016; Mandal et al., 2018). Therefore, we assayed whether impaired retrograde mitochondrial motility impacts mitochondrial calcium buffering using a dual transgenic strategy. We combined two transgenic lines to express G-GECO, a green calcium indicator, in the cytosol (*Tg(5kbneurod:G-GECO)^nl19^*) and R-GECO, a red calcium indicator, in mitochondria (*Tg(5kbneurod:mito-R-GECO)^nl20^;* (Zhao et al., 2011; Mandal et al., 2018). We crossed these transgenes into the *actr10^nl15^* mutant strain and assayed the mitochondrial to cytoplasmic baseline calcium levels in pLL neuronal cell bodies and axon terminals at 4 and 6 dpf (Fig. 3*M–P*). This approach demonstrated that, while there was no difference between *actr10^nl15^* and WT in the neuronal cell body measurements, there was a reduction in mitochondrial:cytosolic calcium levels in mutant axon terminals at both time points (cell body: 4 dpf, *F*_(1,38)_ = 0.488, *p* = 0.489, ANOVA; 6 dpf, *F*_(1,38)_ = 0.0945, *p* = 0.7602, ANOVA; axon terminal: 4 dpf, *F*_(1,39)_ = 5.394, *p* = 0.0255, ANOVA; 6 dpf, *F*_(1,37)_ = 14.196, *p* = 0.0006, ANOVA; Fig. 3*Q*). These results suggest that axon terminal mitochondria have reduced calcium buffering potential when their retrograde transport is inhibited.

**Figure 4.**
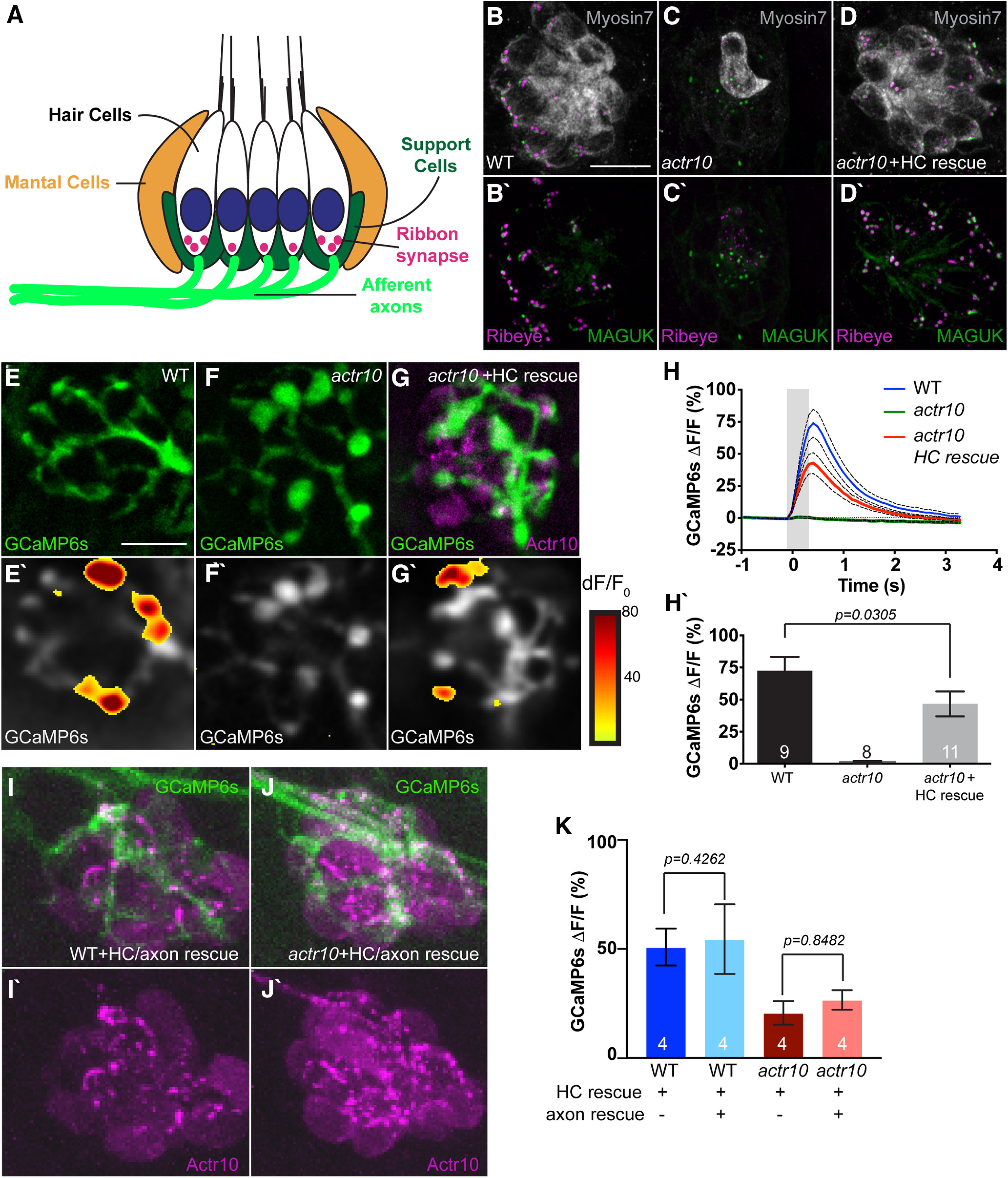
Postsynaptic axon response to stimulation is unaffected by mitochondrial health. ***A***, Schematic of the neural circuit in a sensory neuromast of the pLL. Apical stereocilia on HCs are deflected by water movement and signal through ribbon synapses to afferent (postsynaptic) axons. Support cells surround HCs. ***B***, ***C***, *actr10^nl15^* mutants have fewer sensory HCs as assayed by Myosin VIIa immunolabeling. ***D***, ***D'***, HC and synapse number is rescued by expressing RFP-tagged Actr10 in HCs (HC rescue) using the *Tg(myo6b:mRFP-actr10)^y610^* transgenic. Ribeye: presynapse; MAGUK: postsynapse. ***E–G***, GCaMP6s expression in an axon terminal of a WT (***E***), *actr10^nl15^* mutant (***F***), and *actr10^nl15^* mutant with HC rescue (***G***) at 5 dpf. ***E'–G'***, Shown are spatial patterns of GCaMP6s signal increases in afferent terminals during stimulation. GCaMP6s signals are colorized according to the dF/F_0_ heat maps and are superimposed onto a prestimulus baseline GCaMP6s image. ***H***, ***H'***, The average change in GCaMP6s fluorescence intensity on stimulation, shown in plots (***H***) and quantification (***H'***: WT: 73.26 ± 10.62; *actr10^nl15^*: 1.53 ± 0.76; *actr10^nl15^*+HC rescue: 43 ± 7.78), revealed a reduction in *actr10^nl15^* mutants with and without HC rescue (ANOVA). Black dotted lines represent SEM. ***I***, ***J***, Actr10 rescue in axons using mosaic expression of the *5kbneurod:mRFP-actr10* transgene (axon rescue) in the background of the *Tg(myo6b:mRFP-actr10 ^y610^* transgenic (HC rescue). ***K***, Rescuing Actr10 in neurons does not rescue postsynaptic axonal activity based on average GCaMP6s fluorescence (*t* test). WT: 50.90 ± 8.45; WT+HC rescue: 54.52 ± 16.0; *actr10^nl15^*: 20.69 ± 5.37; *actr10^nl15^*+HC rescue: 26.65 ± 4.45. Sample sizes indicated on graph. Scale bar: 10 µm. All data are mean ± SEM.

Because calcium ion imbalance could affect axonal activity, we asked whether mitochondrial disruptions alter the pLL sensory circuit function. First, we analyzed the synapses made between the presynaptic sensory HCs (Myosin VIIa) and their postsynaptic targets, the pLL axon terminals. Analysis of presynaptic (Ribeye-positive) and postsynaptic (MAGUK-positive) puncta in *actr10^nl15^* mutants and WT siblings somewhat unexpectedly showed that there are virtually no HCs in *actr10^nl15^* mutants (Fig. 4*A–C*). To circumvent this problem, we rescued HCs by creating a transgenic strain that expresses mRFP tagged Actr10 in HCs (*Tg(myo6b:mRFP-actr10)^y610^*). *actr10^nl15^* mutants expressing this rescue transgene (HC rescue) display normal numbers of sensory HCs (WT: 18.73 ± 0.4491; *actr10^nl15^*+HC rescue: 18.23 ± 0.7692; *t*_(22)_ = 0.5313, *p* = 0.6006, *t* test) and complete synapses per HC (WT: 2.661 ± 0.1861; *actr10^nl15^*+HC rescue: 2.349 ± 0.1137; *t*_(22)_ = 1.480, *p* = 0.1530, *t* test) at 5 dpf (Fig. 4*B–D*). Presynapse and postsynapse size were also similar, although there was a slight decrease in presynaptic size (presynapse WT: 0.315 ± 0.0045; *actr10^nl15^*+HC rescue: 0.2954 ± 0.0052; *t*_(14)_ = 0.981, *p* = 0.0067, *t* test; postsynapse WT: 0.2204 ± 0.0059; *actr10^nl15^*+HC rescue: 0.208 ± 0.0048; *t*_(15)_ = 1.181, *p* = 0.1179, *t* test). Using the HC rescue line we then assessed whether *actr10^nl15^* mutants had impaired postsynaptic calcium responses during HC stimulation using previously published protocols (Kindt et al., 2012; Zhang et al., 2016; Lukasz and Kindt, 2018). Using a fluid jet, we mechanically stimulated HCs and monitored the change in GCaMP6s intensity in the pLL axon terminals of *Tg(hsp70l:GCaMP6s-CAAX-SiLL1)^idc8Tg^* animals (Zhang et al., 2018). As expected, *actr10^nl15^* mutants which lack HCs showed no axon terminal response. In *actr10^nl15^* HC rescue animals, we were able to detect responses in axon terminals; however, these responses were significantly smaller than WT controls, indicating that synaptic function was disrupted (*t*_(18)_ = 2.349, *p* = 0.0305, *t* test; Fig. 4*E–H*).

We reasoned that the reduced axon terminal response observed in *actr10^nl15^* HC rescue animals could be cell autonomous, because of mitochondrial dysfunction in the axon, or rather the result of a defect in another component of the synaptic niche (Fig. 4*A*). To determine whether the axonal defect was cell autonomous, we rescued Actr10 in the afferent axons using mosaic expression of mRFP-tagged Actr10 under a neuron specific promotor (axon rescue). We have used this strategy previously to rescue axon morphology and mitochondrial localization in the *actr10^nl15^* mutant (Drerup et al., 2017). The combined axon/HC rescue failed to restore calcium responses in the axon terminals (WT *t*_(6)_ = 0.1999, *p* = 0.8482, *t* test; *actr10 t*_(6)_ = 0.854, *p* = 0.4261, *t* test; Fig. 4*I–K*). This failure to rescue indicates that the decreased postsynaptic axonal response in the *actr10^nl15^* HC rescue animals is not because of a defect in the axon itself.

**Figure 5.**
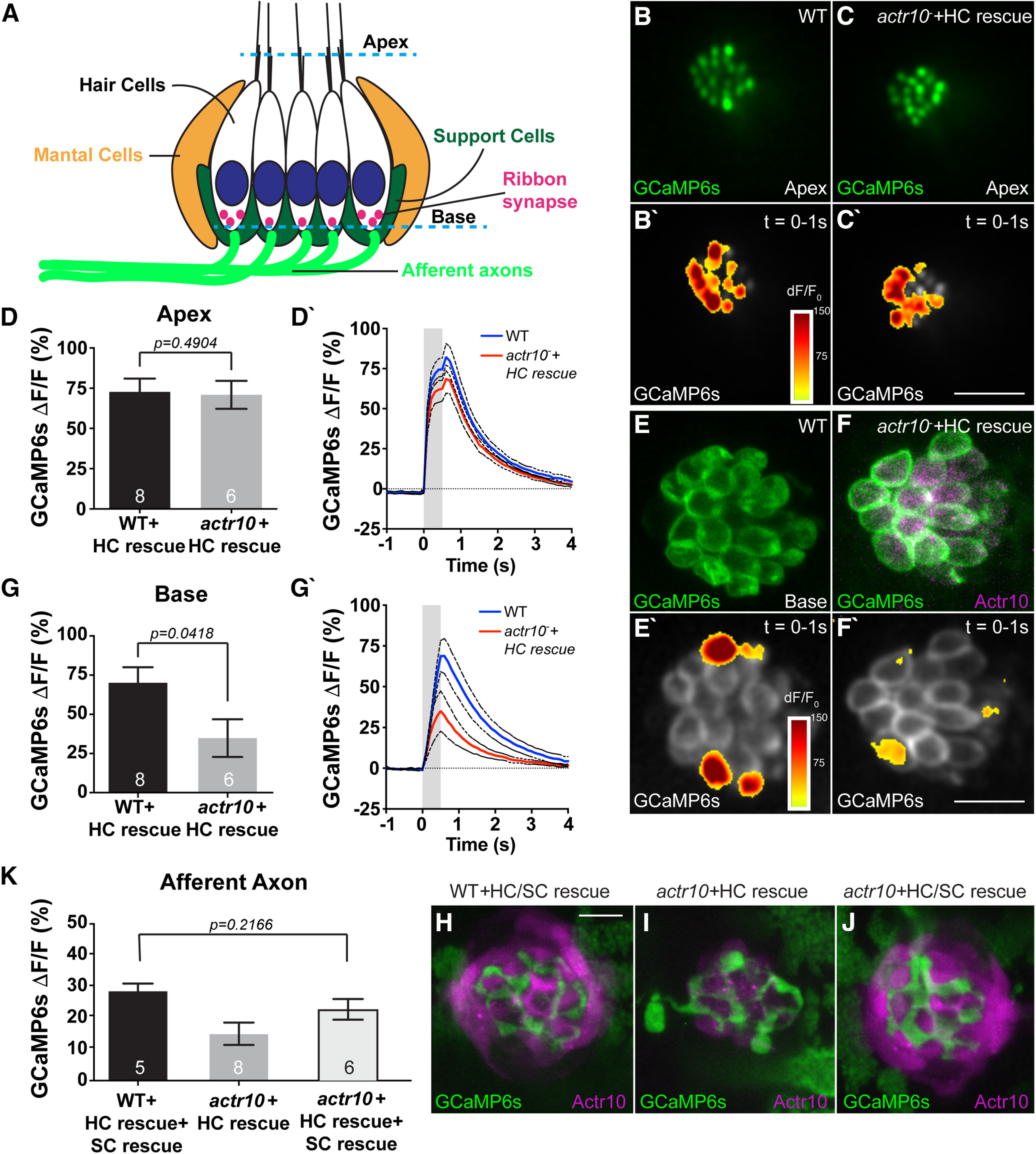
Loss of support cells (SCs) underlies the loss of postsynaptic activity in *actr10^nl15^* mutants. ***A***, Schematic of the neuromast indicating the “apex” of the HC where mechanotransduction takes place in stereocilia and the “base” of the HC where the presynaptic activity occurs. ***B***, ***C***, WT and *actr10^nl15^* mutants with HC rescue at 5 dpf show similar mechanotransduction in HC stereocilia (Apex). ***B***', ***C***', Heat map of Apex GCaMP6s fluorescence intensity changes during stimulation. ***D***, ***D'***, Quantification reveals no difference in the average change in Apex GCaMP6s fluorescence intensity between WT and *actr10^nl15^* mutants with HC rescue (*t* test). WT+HC rescue: 72.73 ± 8.24; *actr10^nl15^*+HC rescue: 70.85 ± 8.71. Black dotted lines represent standard error of the mean. ***E***, ***F***, Compared with WT, *actr10^nl15^* HC rescue mutants show reduced calcium influx at the presynapse. ***E***', ***F***', Heat map of GCaMP6s fluorescence intensity changes at the HC base during stimulation. ***G***, ***G'***, Quantification of the change in Base GCaMP6s fluorescence is significantly different between WT and *actr10^nl15^* mutants with HC rescue (*t* test). WT+HC rescue: 70.09 ± 9.93; *actr10^nl15^*+HC rescue: 34.81 ± 12.05. ***H–J***, Rescue of Actr10 expression in *actr10^nl15^* SCs in the *Tg(She:actr10p2amRFP)^y623^*/*Tg(myo6b:mRFP-actr10)^y610^* double transgenic line. ***H***, WT sibling with HC and SC expression of Actr10. ***I***, *actr10^nl15^* mutant with only HC rescue. ***J***, *actr10^nl15^* mutant with HC/SC double rescue. ***K***, GCaMP6s responses in the axon terminal are normal in *actr10^nl15^* mutant with SC and HC rescue but not with HC rescue alone (ANOVA). WT+HC/SC rescue: 28.05 ± 3.50; *actr10^nl15^*+HC rescue: 17.10 ± 3.08; *actr10^nl15^*+HC/SC rescue: 23.66 ± 3.26. Sample size indicated on graph. Scale bars: 10 µm. All data are mean ± SEM.

To further define the underlying mechanism leading to decreased afferent response, we assayed activity of the sensory HCs using calcium imaging. These experiments were performed as above but with membrane-localized GCaMP6s expressed in the HCs of *Tg(*−*6myo6b:GCaMP6s-CAAX)^idc1Tg^* transgenic animals. Analyses of HC activity at the apex, the site of initial mechanosensory calcium influx in response to HC stimulation, demonstrated normal responses between WT animals and *actr10^nl15^* HC rescue animals (*t*_(4)_ = 0.758, *p* = 0.4904, *t* test; Fig. 5*A–D*). When we analyzed presynaptic activity at the HC base, however, we noted a 50% reduction in calcium flux, indicating that presynaptic release may be impaired (*t*_(12)_ = 2.278, *p* = 0.0418, *t* test; Fig. 5*A*,*E–G*). These data suggest that another component of the sensory organ is impaired in *actr10^nl15^* mutants. One candidate cell type of interest in this organ are the glia-like supporting cells (SCs) that surround HCs. Recently, these cells have been proposed as a modulator of presynaptic activity in HCs, making them a good candidate for contributing to impaired function in *actr10^nl15^* mutants (Zhang et al., 2018). Therefore, we generated a SC rescue line in which Actr10 and cytoplasmic mRFP are co-expressed in this cell type (*Tg(She:actr10p2amRFP)^y623^*). In the SC/HC double-rescue transgenic, despite a lack of Actr10 in the afferent neurons, postsynaptic responses were at WT levels (*q*_(23)_ = 2.443, *p* = 0.2166, ANOVA with Tukey's HSD; Fig. 5*J*,*K*). This result reiterates the importance of SC function on regulation of HC activity. Furthermore, it suggests that the maintenance of mitochondrial health in sensory axon terminals has little effect on the function of the postsynaptic afferent axon terminal.

### Motor neuron synaptic vesicle release and swimming behavior are disrupted in *actr10^nl15^* mutants

**Figure 6.**
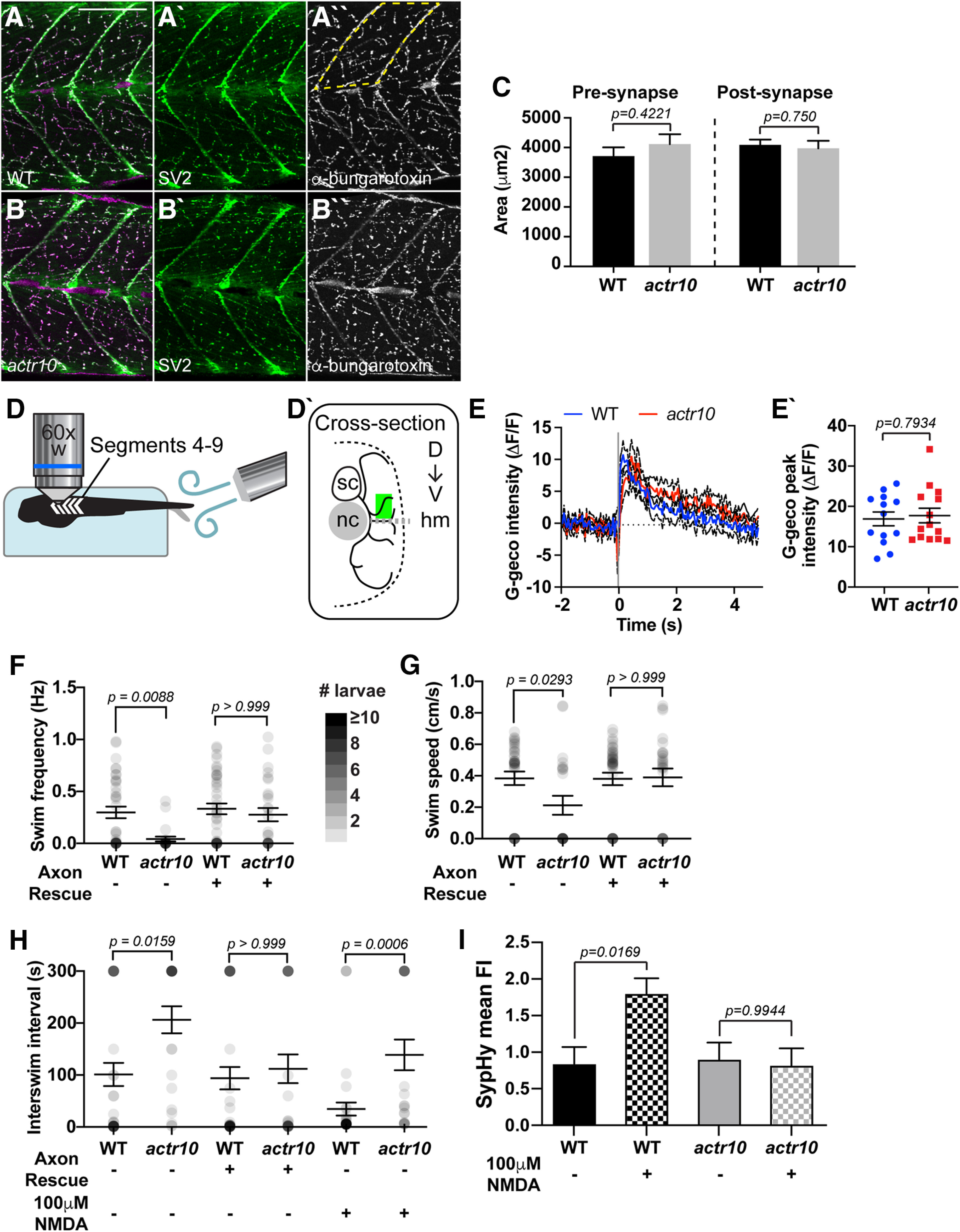
Motor neuron function is decreased in *actr10^nl15^* mutants. ***A***, ***B***, Antibody labeling of larval neuromuscular junctions at 4 dpf revealed no difference between WT (***A***) and *actr10^nl15^* mutants (***B***). ***C***, Quantification of total presynaptic and postsynaptic area in dorsal somite (area outlined by dotted line in ***A''***). Presynapse WT: 3705 ± 390; *actr10^nl15^*: 4114 ± 302. Postsynapse WT: 4088 ± 285; *actr10^nl15^*: 3971 ± 220. ***D***, Schematic of motor neuron axon calcium imaging. Larvae are paralyzed and immobilized in agarose with tail freed for stimulation and randomly oriented such that either left or right side of body segments 4–9 faces the objective. ***D'***, Cross-section of recording area. Motor neuron (black lines) expressing cell-fill G-Geco originate from the spinal cord (sc). Motor neuron terminals were randomly selected in a region (green rectangle) dorsal to the horizontal myoseptum (gray dotted line; hm) and lateral to the notochord (nc). ***E***, Average temporal traces of evoked terminal G-Geco signals (ΔF/F_0_). Black dotted lines represent SEM. ***E'***, Scatterplot of peak terminal-Ca^2+^ response in WT (blue) or *actr10^nl15^* mutants (red) show no change in response in mutants. WT: 16.9 ± 1.726; *actr10^nl15^*: 17.72 ± 1.815. ***F–H***, Dot plots show spontaneous swim frequency, speed, and time interval between swim bouts, respectively. Dot intensity (grayscale) corresponds to number of larvae at each point with darker values indicating more larvae displayed the behavior. *actr10^nl15^* mutants swim less frequently and are slower than WT siblings. These behavioral deficits are rescued with neuronal Actr10 expression. Frequency, WT: 0.2986 ± 0.056; *actr10^nl15^*: 0.042 ± 0.023; WT+Axon rescue: 0.333 ± 0.052; *actr10^nl15^*+Axon rescue: 0.277 ± 0.063. Speed, WT: 0.384 ± 0.043; *actr10^nl15^*: 0.212 ± 0.061; WT+Axon rescue: 0.380 ± 0.040; *actr10^nl15^*+Axon rescue: 0.390 ± 0.056. Interswim interval, WT: 101 ± 22.31; *actr10^nl15^*: 206 ± 26.05; WT+Axon rescue: 93.89 ± 21.39; *actr10^nl15^*+Axon rescue: 112 ± 27.68. ***H***, NMDA treatment decreases interswim intervals but *actr10^nl15^* mutants still swim less frequently than WT siblings. WT: 30.64 ± 12.68; *actr10^nl15^*: 136 ± 29.97. ***I***, SypHy mean fluorescence (normalized to mRFP fill); 100 μm NMDA increases SypHy fluorescence in WT but not *actr10^nl15^* mutants, indicating no increase in synaptic vesicle release. WT: 0.84 ± 0.233; WT+NMDA: 1.80 ± 0.214. *actr10^nl15^*: 0.901 ± 0.219; *actr10^nl15^* +NMDA: 0.821 ± 0.219. Scale bars: 100 µm. All data are mean ± SEM.

The lack of defective pLL axon responses in our *actr10^nl15^* mutant axon terminals does not align with the observed defects in axonal activity when mitochondria are disrupted in hippocampal neurons (Rangaraju et al., 2014). We reasoned that this was perhaps because of the fact that pLL axon terminals are postsynaptic and do not have the energetic demands associated with vesicle cycling/recruitment that presynaptic axons do. To determine whether the mitochondrial dysfunction observed in *actr10^nl15^* mutant axons affected presynapse function, we analyzed motor neuron axon structure and function in this line. Work on the *Drosophila* neuromuscular junction has shown that this synapse relies heavily on ATP-driven vesicle recruitment with stimulation (Verstreken et al., 2005). First, we analyzed general presynapse and postsynapse formation in the trunk of 4 dpf larvae. Staining for presynaptic and postsynaptic markers in the axons and muscle, respectively, revealed no change in the total area of either synaptic component in a segment of the mid-trunk of *actr10^nl15^* mutants (segment analyzed outlined; presynapse, *F*_(1,14)_ = 0.6839, *p* = 0.4221, ANOVA; postsynapse, *F*_(1,14)_ = 0.106, *p* = 0.750, ANOVA; Fig. 6*A–C*). We then asked whether motor neuron axon function was altered in *actr10^nl15^* mutants using three separate sets of experiments: calcium imaging in the axon terminal, swimming behavior, and imaging of spontaneous synaptic vesicle release. First, we imaged evoked presynaptic calcium influx at axon terminals, similar to our analyses in the pLL sensory system. For this, WT and *actr10^nl15^* mutant larvae carrying the *Tg(5kbneurod:G-GECO)^nl19^* transgene were immobilized in agarose and a section was removed to expose the tail. The tail was then physically deflected to elicit a fictive escape, while the larvae were held stationary by the agarose (Fig. 6*D*). Before, during, and after stimulation, a motor neuron axon terminal in the mid-trunk was imaged and the change in G-Geco fluorescence analyzed (Fig. 6*D'*). Analysis of G-Geco fluorescence revealed similar changes in calcium in axon terminals between WT and *actr10^nl15^* mutants (*t*_(25)_ = 0.2647, *p* = 0.7934, *t* test; Fig. 6*E*,*E'*).

This result suggests that mitochondrial dysfunction in axon terminals does not alter calcium dynamics. Calcium influx is upstream of synaptic vesicle release. Because previous work suggested that both synaptic vesicle cycling and synaptic vesicle recruitment depend on mitochondrial function (Verstreken et al., 2005; Rangaraju et al., 2014), we next asked whether neurotransmission was intact by analyzing swimming behavior. Spontaneous swimming was recorded in WT and *actr10^nl15^* mutants over 5 min. Analysis of swimming frequency, swim speed, and interswim interval revealed changes in *actr10^nl15^* mutants (frequency *Z* = 3.182, *p* = 0.0088; speed *Z* = 2.814, *p* = 0.0293; interswim interval *Z* = 3.274, *p* = 0.0159; Kruskal–Wallis with Dunn's multiple comparisons; Fig. 6*F–H*). This could be because of a defect in the motor neurons or could also be attributed to unidentified defects in the muscle. To determine whether neuronal abnormalities were the cause of the swimming defects, we analyzed swimming behavior in WT and *actr10^nl15^* animals carrying the *Tg(5kbneurod:mRFP-actr10)^nl22^* transgene (axon rescue). This transgene rescues Actr10 expression in neurons, including motor neurons. *actr10^nl15^* mutants with axon rescue displayed normal swimming behavior, confirming that neuronal defects underly this behavioral phenotype (frequency *Z* = 0.6943, *p* > 0.9999; speed *Z* = 0.1212, *p* > 0.9999; interswim interval *Z* = 0.7279, *p* > 0.9999; Kruskal–Wallis with Dunn's multiple comparisons; Fig. 6*F–H*). Finally, we specifically addressed the impact of abnormal mitochondrial function in *actr10^nl15^* mutants on synaptic vesicle release using SypHy, a pH-sensitive version of GFP targeted to synaptic vesicles (Odermatt et al., 2012; Zhang et al., 2018). SypHy was co-expressed with mRFP in motor neurons under the *mnx1* promotor and spontaneous vesicle release was imaged over 5 min. This revealed no difference in average SypHy fluorescence between WT and *actr10^nl15^* mutants (*q* = 2.675, *p* = 0.9976, Tukey–Kramer HSD; Fig. 6*I*). We then wanted to challenge the system by increasing vesicle release which increases vesicle cycling and recruitment. Both vesicle cycling and recruitment have been shown to rely on mitochondrial-dependent ATP production (Verstreken et al., 2005; Rangaraju et al., 2014). For this we augmented activity in this circuit using bath application of 100 μm NMDA, which has been shown to increase spontaneous swimming activity in zebrafish larvae (Cui et al., 2004; Low et al., 2011). We first confirmed that application of NMDA decreases the interswim interval in WT animals (*U* = 471.5, *p* = 0.0145, Mann–Whitney; Fig. 6*H*). We then performed analyses of SypHy fluorescence in motor neuron axons after application of 100 μm NMDA. This revealed a significant increase in fluorescence in WT animals, indicating increased spontaneous synaptic release, but no change in *actr10^nl15^* mutants (model *q* = 2.675, *p*_(WT)_ = 0.0169, *p*_(_*_actr10nl15_*_)_ = 0.9944; Tukey–Kramer HSD; Fig. 6*I*). Together, our behavioral and SypHy data indicate that *actr10^nl15^* mutants have defective motor neuron synaptic transmission.

### Complete turnover of axon terminal mitochondria occurs within 24 h

**Figure 7.**
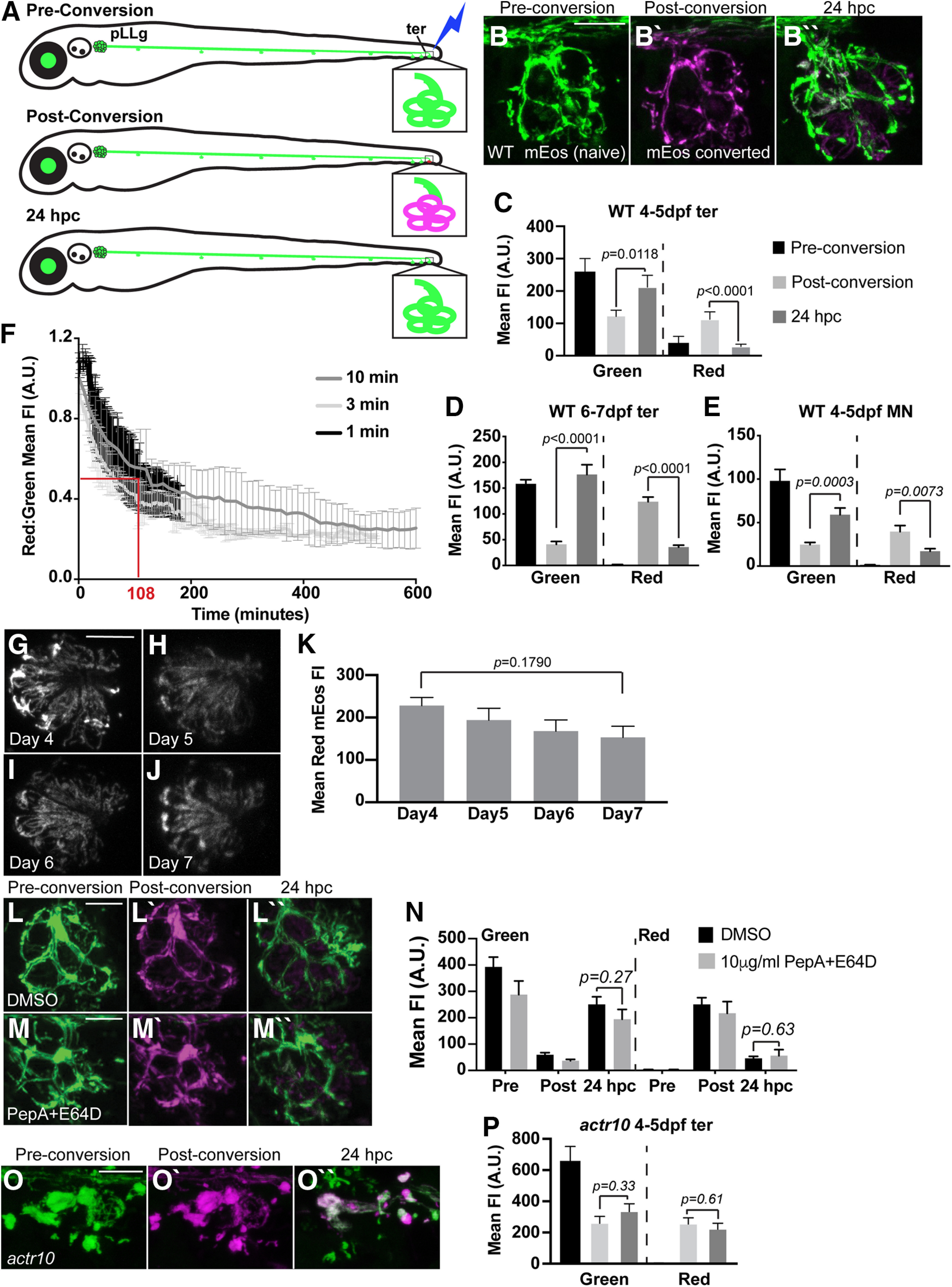
Mitochondrial retrograde transport is required for mitochondrial turnover in axon terminals. ***A***, Schematic of mitochondrial photoconversion assay using the *Tg(5kbneurod:mito-mEos)^y568^* transgenic zebrafish. pLL axon terminals of the terminal cluster (ter) are depicted in the inset. ***A***, ***B***, Photoconversion of mitochondria in a WT pLL axon terminal results in a permanent switch from green to red (red is shown in magenta) of the mitochondrially localized mEos (photoconversion at 4 dpf). These converted mitochondria are gone 24 h postphotoconversion (hpc). ***C***, Quantification of the gain of new (green) and loss of old (magenta) mitochondria from axon terminals 24 hpc (ANOVA; Tukey's HSD *post hoc* contrasts; *n* = 8 each). Green pre: 252.12 ± 31.44; green post: 103.99 ± 13.55; green 24 hpc: 183.05 ± 26.04; red pre: 23.50 ± 13.59; red post: 90.79 ± 15.20; red 24 hpc: 20.80 ± 4.93. ***D***, pLL axon terminal mitochondria at 6 dpf show similar levels of mitochondrial turnover to that observed at 4 dpf (ANOVA; *n* = 21). Green pre: 155.06 ± 8.14; green post: 39.78 ± 4.74; green 24 hpc: 173.50 ± 21.87; red pre: 1.67 ± 0.15; red post: 123.63 ± 9.04; red 24 hpc: 35.56 ± 3.85. ***E***, Motor neuron axons show similar levels of mitochondrial turnover compared with pLL sensory axons in 24 h (ANOVA; *n* = 13). Green pre: 102.58 ± 13.38; green post: 25.69 ± 2.50; green 24 hpc: 62.69 ± 7.37; red pre: 1.41 ± 0.15; red post: 42.16 ± 7.04; red 24 hpc: 18.31 ± 2.90. ***F***, Time-lapse imaging of mitochondrial turnover in pLL axon terminals reveals that 50% of mitochondria have left the axon terminal by 108 min postconversion (1 min: *n* = 4; 3 min: *n* = 2; 10 min: *n* = 2). ***G–K***, Photoconversion of mEos-labeled mitochondria in HCs of the pLL showed no significant loss of red mEos until 72 hpc (ANOVA with *post hoc* contrasts; *n* = 12). Day 4: 228 ± 19.26; day 5: 193.98 ± 27.91; day 6: 167.95 ± 26.35; day 7: 153.24 ± 26.01. ***L***, ***M***, Photoconversion of axon terminal mitochondria followed by treatment with lysosomal inhibitors pepstatin A and E64D (10 µg/ml for ∼18 h) did not impair mitochondrial turnover in pLL axon terminals. ***N***, Quantification of mitochondrial turnover with lysosomal inhibition (ANOVA; *n* = 11). DMSO control: green pre: 391.75 ± 32.26; green post: 61.61 ± 5.98; green 24 hpc: 239.13 ± 20.72; red pre: 5.02 ± 1.16; red post: 269.86 ± 28.07; red 24 hpc: 46.24 ± 5.44. Pepstatin A/E64D: green pre: 287.63 ± 51.72; green post: 36.88 ± 4.85; green 24 hpc: 194.22 ± 37.30; red pre: 1.92 ± 0.21; red post: 216.81 ± 44.20; red 24 hpc: 55.76 ± 23.12. ***O***, Photoconversion of mitochondrially-localized mEos in a pLL axon terminal of an *actr10^nl15^* mutant. ***P***, Quantification of new (green) and old (magenta) mitochondria shows persistence of converted mitochondria in pLL axon terminals when retrograde transport is disrupted (ANOVA; Tukey's HSD *post hoc* contrasts; *n* = 6). Green pre: 651.94 ± 104.49; green post: 248.06 ± 51.95; green 24 hpc: 325.04 ± 55.11; red pre: 5.12 ± 1.05; red post: 243.56 ± 47.47; red 24 hpc: 210.60 ± 42.90. Pre, before conversion; post, immediately after conversion. Scale bars: 10 µm. All data are mean ± SEM.

Our analyses of mitochondrial localization and health indicates that retrograde transport is important for distribution and maintenance of the population. This made us curious about the frequency of retrograde movement of the axon terminal mitochondrial population. Imaging of mitochondrial axonal transport has typically been examined on the order of minutes in cultured neurons and, more recently, *in vivo* in mouse brain and sciatic nerve (Misgeld et al., 2007; Lewis et al., 2016). Additionally, this imaging has traditionally been done along the axon shaft rather than in the terminal. These studies have not analyzed the frequency of mitochondrial movement in axons over hours, and, more specifically, movement of the mitochondrial population in the axon terminal. To directly test whether mitochondria are stationary in axon terminals over hours and days, we engineered a transgenic zebrafish (*Tg(5kbneurod:mito-mEos)^y568^*) to label the mitochondrial intermembrane space in pLL neurons with the photoconvertible protein mEos (Mandal et al., 2018). We photoconverted mitochondrial mEos in the distal axon terminals (ter) at 4 dpf and measured green (native) and red (photoconverted) mEos fluorescence intensities in the converted compartments immediately after conversion and 24 hpc. For this analysis, we used the same confocal laser power and detector settings immediately postconversion and 24 hpc. Quantification of fluorescence intensities revealed addition of new (green) mitochondria and almost complete loss of the photoconverted (red) mitochondria from axon terminals in this time-period, indicating that there is significant organelle turnover within a day (green, *F*_(1,28)_ = 7.255, *p* = 0.0118, ANOVA; red, *Z* = 4.189, *p* < 0.0001, Wilcoxon; Fig. 7*A–C*).

To determine whether there was a residual developmental component to this mitochondrial turnover between 4 and 5 dpf, we performed the same experiment at a slightly later developmental time point. Similar to what we observed previously, the mitochondrial axon terminal populations showed complete turnover between 6 and 7 dpf in pLL axons (green, *Z* = −4.480, *p* < 0.0001, Wilcoxon; red, *Z* = −4.480, *p* < 0.0001, Wilcoxon; Fig. 7*D*). Next, we wanted to determine whether this rapid mitochondrial turnover also occurred in motor neurons axons. We photoconverted the distal motor neuron axon at 4 dpf and analyzed mEos fluorescence intensities at 5 dpf. This analysis revealed a similar mitochondrial turnover in the motor neuron axonal population (green, *Z* = −3.608, *p* = 0.0003, Wilcoxon; red, *Z* = 2.685, *p* = 0.0073, Wilcoxon; Fig. 7*E*). These experiments revealed that mitochondrial turnover occurs consistently within 24 h at multiple developmental stages and in both sensory and motor neuron axon terminals *in vivo*.

We then asked precisely how long it takes for mitochondria to leave the axon terminal using high resolution time-lapse imaging immediately after photoconversion. With inter-image intervals of 1, 3, or 10 min, we found that 50% of the converted mitochondria were depleted from pLL sensory axon terminals 108 min after conversion (Fig. 7*F*). Together, our data show that reliable mitochondrial turnover occurs in sensory and motor neuron axons on the scale of hours.

### Mitochondria redistribute from axon terminals throughout the neuron via retrograde transport

The axon terminal mitochondrial turnover was rapid. Therefore, we wanted to confirm that our measurements reflected actual mitochondrial exit from the axon terminal rather than decay of the mitochondrially localized mEos. To quantify mEos decay, we took advantage of the expression of this protein in mitochondria of HCs (sensory cell of the pLL) in this transgenic. This is the only non-neuronal cell that expresses mitochondrially localized mEos in the *Tg(5kbneurod:mito-mEos)^y568^* transgenic. Unlike in neurons where mitochondria can move freely between neuronal compartments, in HCs mEos is confined within these flask-like, easily definable, cells. Quantification of mitochondrially localized, photoconverted mEos in HCs revealed that mEos decay cannot account for the mitochondrial turnover observed in pLL axon terminals (*F*_(3,44)_ = 1.7088, *p* = 0.1790, ANOVA; Fig. 7*G–K*).

Mitochondrial turnover in axon terminals could also be because of local degradation of the organelle. Indeed, some local mitochondrial mitophagy has been observed in distal axons (Ashrafi et al., 2014). To determine whether mitophagy contributed to the loss of mitochondria from axon terminals, we photoconverted mitochondria marked by mEos, treated animals with lysosomal inhibitors (Tanida et al., 2005; He et al., 2009), and analyzed mitochondrial turnover as above. No change in the turnover of mitochondria in axon terminals following lysosomal inhibition was observed (green, *F*_(1,26)_ = 1.298, *p* = 0.2650, ANOVA; red, *F*_(1,26)_ = 0.2355, *p* = 0.6315, ANOVA; Fig. 7*L–N*), indicating that local mitophagy in axon terminals cannot account for the observed mitochondrial turnover.

As local mitophagy and mEos bleaching could not explain the observed turnover of mitochondria in axon terminals, we wanted to determine the contribution of retrograde transport to clearance of the mitochondrial population. For this, we again used the *actr10^nl15^* mutant strain in which retrograde mitochondrial transport is disrupted (Drerup et al., 2017). First, we photoconverted the entire axon terminal and analyzed red (old) and green (new) mitochondria 24 hpc as above (see Fig. 7*A–C*), using the same imaging settings at 4 and 5 dpf. There was no loss of red fluorescence in the *actr10^nl15^* mutant line at 5 dpf, indicating that disrupting retrograde mitochondrial movement eliminates turnover from the axon terminal (green, *F*_(1,14)_ = 1.0331, *p* = 0.3267, ANOVA; red, *F*_(1,14)_ = 0.2653, *p* = 0.6145, ANOVA; Fig. 7*O*,*P*). Notably, anterograde mitochondrial transport is intact in the *actr10^nl15^* mutant line (Drerup et al., 2017), and we do observe increased green fluorescence 24 hpc, but this does not reach statistical significance. The upward trend in green fluorescence perhaps reflected a low rate of new organelle addition because of overcrowding at the axon terminal.

**Figure 8.**
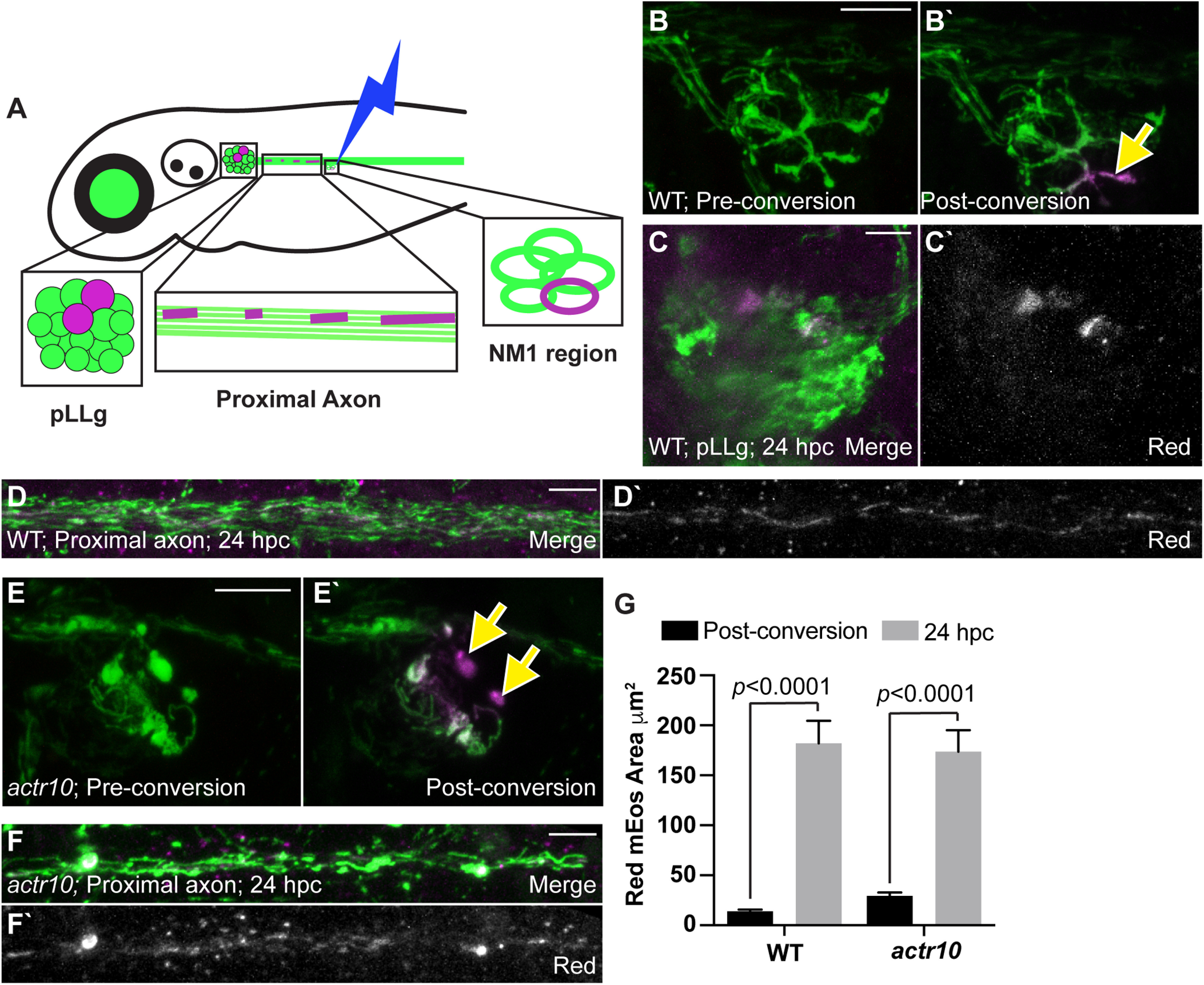
mEos can be redistributed independent of retrograde transport in pLL neurons. ***A***, Schematic of the minimal mitochondrial photoconversion strategy and the regions analyzed for mitochondrial area at 24 hpc. ***B***, Images of a WT axon terminal before and immediately after photoconversion. Green: naive mEos; magenta: converted mEos. Arrow points to region photoconverted. ***C***, ***D***, The pLLg and proximal axon at 24 hpc shows mitochondrially localized converted (magenta in merge, white alone) mEos in the cell body and mitochondria along the axon. ***E***, An *actr10^nl15^* mutant axon terminal before and immediately after photoconversion. ***F***, The proximal axon of an *actr10^nl15^* mutant 24 hpc showing converted mEos in mitochondria in the axon. ***G***, Quantification of the total area of converted mEos fluorescence immediately postconversion and at 24 hpc (ANOVA; WT: *n* = 9; *actr10^nl15^*: *n* = 13). Postconversion: WT: 14.16 ± 2.97; *actr10^nl15^*: 29.52 ± 2.60. 24 hpc: WT: 181.98 ± 23.70; *actr10^nl15^*: 173.55 ± 20.79. Scale bars: 10 µm. All data are mean ± SEM.

This failed mitochondrial turnover in *actr10^nl15^* mutants could be due directly to a role for retrograde mitochondrial transport in movement of this organelle population or a result of failed fission/fusion dynamics of mitochondria in the *actr10^n15^* mutant line. To address this, we assessed the impact of mitochondrial fission/fusion dynamics to the redistribution of converted mitochondria. Because of the dense nature of the mitochondria in the axon, we could not definitively observe individual mitochondrial fission and fusion events. To indirectly assess mitochondrial dynamics, we photoconverted a small region of WT axon terminals at 4 dpf, converting as small a mitochondrial area possible, and calculated the change in total converted (red) mEos area at 5 dpf throughout the entire neuron. To detect even the smallest visible change in red fluorescence at 5 dpf, we maximized the detector sensitivity and laser power used and analyzed confocal *z*-stacks through overlapping regions. We reasoned that increased area of red fluorescence would provide insight into mEos spread because of mitochondrial dynamics. Analysis of the change in total area of red fluorescence revealed a significant increase between 4 and 5 dpf (*Z* = 3.742, *p* < 0.0001, Wilcoxon; Fig. 8*A–D*,*G*), suggesting that mitochondrial dynamics can redistribute converted mEos from axon terminal mitochondria throughout the population; however, we observed similar spread of red fluorescence after conversion in the *actr10^nl15^* mutant, indicating that mitochondrial dynamics are likely intact in this line (*Z* = 4.256, *p* < 0.0001, Wilcoxon; Fig. 8*E–G*). Together, these experiments demonstrate that mitochondrial dynamics can redistribute some mEos protein from axon terminal mitochondria throughout the population, but retrograde transport is the primary mediator of organelle turnover in axon terminals.

**Figure 9.**
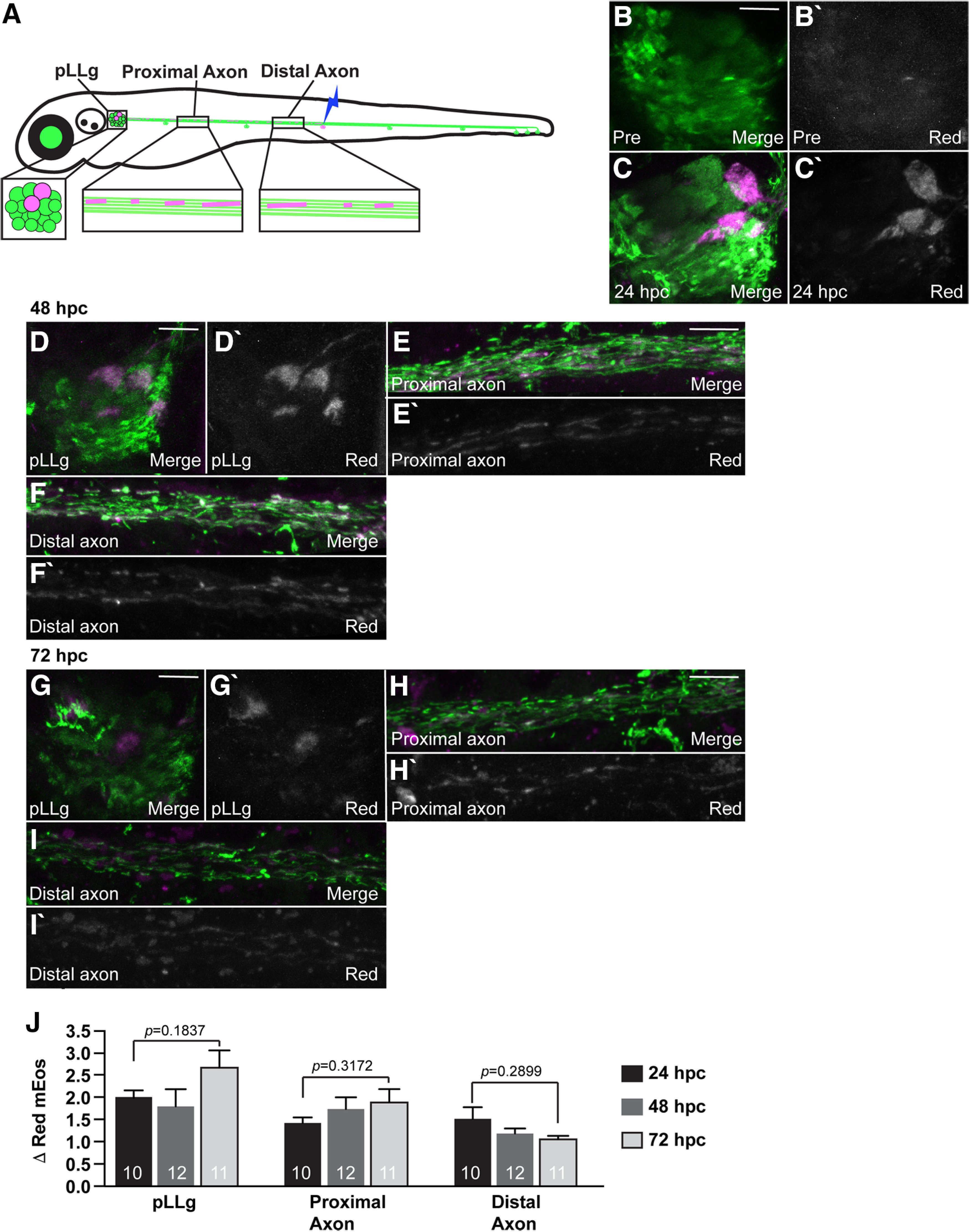
Mitochondrially localized mEos persists for days in neurons. ***A***, Schematic of the photoconversion and tracking strategy. At 4 dpf, pLL axon terminal mitochondria in the NM3 sensory organ (mid-trunk) were photoconverted. At 24, 48, and 72 hpc, the pLLg, proximal axon, and distal axon were imaged. ***B***, ***C***, 24 hpc, mitochondrially localized, converted mEos that originated from the axon terminal is now redistributed to the neuronal cell bodies (magenta or white). We expect two to four neurons have axon terminals in this region at 4 dpf. ***D–F***, 48 hpc, mitochondria converted in NM3 axon terminals are present in the associated cell bodies, proximal, and distal axons of the neuron. ***G–I***, Similarly, 72 hpc mitochondria labeled in the NM3 axon terminals are present throughout the neurons from the cell bodies to the distal axon. ***J***, Quantification of the change in red (converted) fluorescence intensity shows that mitochondrially localized mEos persists in the neuron through 72 hpc (*n* = 12 larvae each; ANOVA; Tukey's HSD *post hoc* contrasts). pLLg: 24 hpc: 2.10 ± 0.18; 48 hpc: 1.81 ± 0.38; 72 hpc: 2.69 ± 0.38. Proximal axon: 24 hpc: 1.37 ± 0.16; 48 hpc: 1.75 ± 0.26; 72 hpc: 1.91 ± 0.29; distal axon: 24 hpc: 1.21 ± 0.11; 48 hpc: 1.20 ± 0.09; 72 hpc: 1.08 ± 0.01. Scale bars: 10 µm. All data are mean ± SEM.

The frequency of mitochondrial retrograde transport and the dramatic change to the distribution of the population of this organelle when this process is disrupted (see Fig. 1) led us to ask what happens to these organelles over time when they move out of the axon terminal. To answer this question, we photoconverted mitochondria in two mid-trunk axon terminals that end at the NM3 sensory organ at 4 dpf. We then tracked the location of these mitochondria in these two axons 24, 48, and 72 h later. For this, we imaged three distinct regions of the pLL: (1) the neuronal cell bodies in the pLL ganglion (pLLg); (2) axons in the proximal pLL nerve; and (3) axons of the distal pLL nerve immediately rostral to the photoconverted terminals (Fig. 9*A*). One day after photoconversion, converted (red) mitochondria were visible in two pLL neuronal cell bodies connected to the two axon terminals photoconverted the previous day (Fig. 9*B*,*C*). When we imaged 48 and 72 hpc, we continued to see converted organelles in all neuronal compartments imaged of the two neurons whose axon terminals had been photoconverted (pLLg, *F*_(2,30)_ = 1.794, *p* = 0.1837, ANOVA; proximal axon, *F*_(2,30)_ = 1.193, *p* = 0.3172, ANOVA; distal axon, *F*_(2,30)_ = 1.291, *p* = 0.2899, ANOVA; Fig. 9*D–J*). These experiments revealed the consistent presence of converted mitochondria throughout the neurons over the 3-d analysis period, suggesting that mitochondria which are retrogradely transported from axon terminals are not degraded but rather persist to be redistributed throughout the neuron.

**Figure 10.**
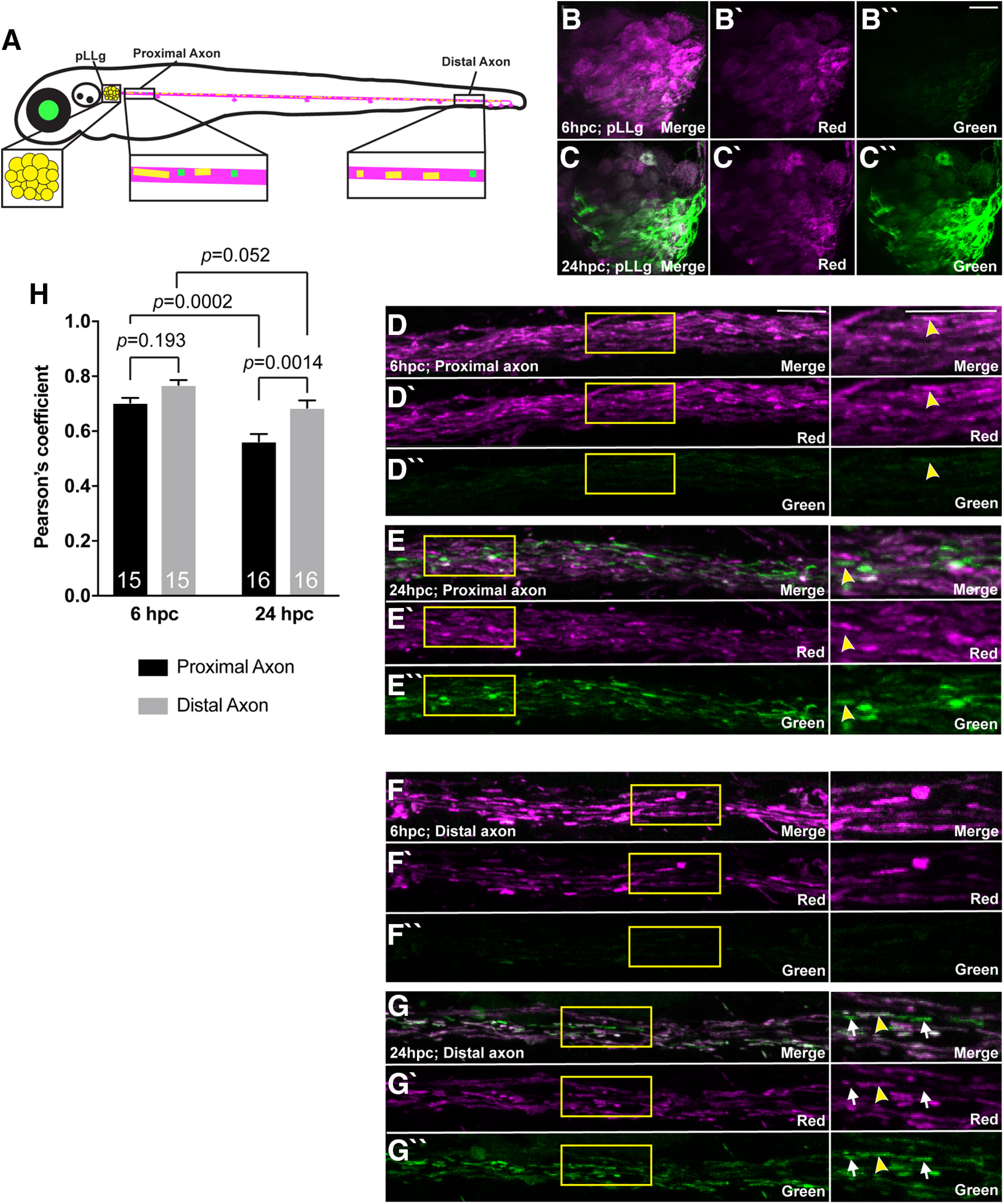
A strong correlation between red and green mEos 24 hpc suggests protein mixing within existing organelles. ***A***, Schematic of the photoconversion and tracking assay. At 4 dpf, the whole larva was photoconverted, and then 6 and 24 hpc, the boxed regions were imaged. ***B–G***, Imaging at 6 and 24 hpc of the whole larva shows gradual gain of new (green) mEos in the cell bodies of the pLLg (***B***, ***C***), proximal axon (***D***, ***E***), and distal axons (***F***, ***G***) of the pLL. For ***D–G***, boxed region is shown at a higher magnification on the right. Arrowheads point to mitochondria with strong green fluorescence and consistent red (converted) fluorescence as well. Arrows point to mitochondria that lack converted mEos. ***H***, Correlational analysis of green and red mEos at 6 and 24 hpc (ANOVA with Tukey's HSD *post hoc* contrasts; *n* = 8). Green signal was converted into a mask to assay the presence of red (converted) fluorescence that overlapped. Proximal axon: 6 hpc: 0.70 ± 0.01; 24 hpc: 0.56 ± 0.02; Distal axon: 6 hpc: 0.77 ± 0.02; 24 hpc: 0.69 ± 0.03. Scale bar: 10 µm. Sample size on graph. All data are ±SEM.

The persistence of mitochondria originally labeled in axon terminals over several days led us to ask how much mitochondrial biogenesis was occurring in sensory neurons *in vivo*. To assay the extent of *de novo* mitochondrial addition, we again used photoconversion but, this time, photoconverted the entire larvae at 4 dpf and analyzed the addition of new (green) organelles 6 and 24 h later (Fig. 10*A*). Although very little green mEos was visible 6 hpc (Fig. 10*B*,*D*,*F*); by 24 hpc, a strong green mEos signal was observed (Fig. 10*C*,*E*,*G*). We then asked whether these green (new) organelles contained any red mEos. The presence of only unconverted (green) mEos would suggest *de novo* biogenesis rather than comingling of existing mitochondrial populations with new protein and/or organelles. For this analysis, we used the green mitochondrial signal as a mask in ImageJ and asked whether red mEos was present in this population using colocalization analysis (Bolte and Cordelières, 2006). We observed a high correlation between red and green mEos in this population at the 6 and 24 h time point in the proximal and distal axon implying that new (green) mEos was being loaded into old (red) mitochondria either through protein reloading or mitochondrial fusion. Some organelles with only unconverted mEos were present as well, and they increased between 6 and 24 hpc. This is evident through the decreased correlation coefficients between 6 and 24 hpc (model *q* = 2.645, *p*_(6 hpc)_ = 0.193, *p*_(24 hpc)_ = 0.0014, *p*_(proximal axon)_ = 0.0002, *p*_(distal axon)_ = 0.052; Tukey–Kramer HSD; Fig. 10*H*). These data support a model in which both mitochondrial biogenesis in the cell body and replenishment of existing organelles contributes to a healthy mitochondrial population in the neuron.

Together, our work implicates retrograde mitochondrial transport in the support of an evenly distributed, healthy population of mitochondria in neurons. Disruption of this process causes loss of cell body mitochondria, accumulation of unhealthy organelles in axon terminals, and impacts presynaptic function in motor neuron axons. Photoconversion and tracking experiments suggest that this population is supported through mitochondrial biogenesis as well as mitochondrial repair through protein reloading and mitochondrial fission and fusion. However, these processes are not sufficient to support axon terminal organelles in the absence of retrograde transport.

## Discussion

The necessity of mitochondrial transport in neurons is demonstrated by the wealth of clinical literature showing a correlation between abnormal mitochondrial localization and neurodegenerative disease. For anterograde transport, the reasons for pathology are intuitive as anterograde mitochondrial transport is essential for moving organelles into the axon from the primary site of mitochondrial biogenesis near the nucleus (Davis and Clayton, 1996). The purpose of retrograde mitochondrial movement has been shown to be the removal of damaged organelles from the axon for degradation. This conclusion is based on pharmacological manipulations that perturb mitochondrial health and lead to enhanced retrograde mitochondrial transport. For example, treatment of cultured neurons with drugs such as rotenone, antimycin A, FCCP, and others cause a loss of mitochondrial matrix potential and can lead to enhanced retrograde mitochondrial motility (Miller and Sheetz, 2004; Ashrafi et al., 2014; Lin et al., 2017). Additionally, analyses of mitochondrial movement in neurons treated with a matrix potential indicator have shown that lower matrix potentials correlate with retrograde movement (Miller and Sheetz, 2004; Ashrafi et al., 2014; Lin et al., 2017), although this is somewhat controversial in the field (Verburg and Hollenbeck, 2008; Suzuki et al., 2018). Together, these studies imply that mitochondrial health feeds into a larger signaling network that regulates mitochondrial transport and localization.

Our work supports this model as disruption of retrograde movement causes an accumulation of damaged organelles in the distal axon. Additionally, our work also shows that the consistent retrograde mitochondrial movement is a normal process in healthy neurons. This movement helps to not only prevent accumulation of damaged organelles in the distal axon but is also essential to maintain a homeostatic distribution of healthy mitochondria throughout the neuron's large volume. By imaging neuronal mitochondrial localization over time scales of hours and days, we have shown that retrograde mitochondrial motility occurs in axons at a reliable rate. Specifically, in synaptically active axon terminals of WT animals, we observed complete turnover of the axon terminal mitochondrial population in <24 h as a physiologically normal component of neuronal function. Importantly, these results were obtained in a completely intact, *in vivo* system, without pharmacological manipulation of mitochondrial health. Our results indicate that retrograde mitochondrial motility occurs and is necessary in the absence of overt mitochondrial damage to maintain a balanced and healthy organelle population. Furthermore, these organelles appear to at least in part persist for days after they leave the axon terminal. This persistence argues that retrograde mitochondrial transport is not just a disposal mechanism for the removal and ultimate degradation of this organelle from axons. Rather, these organelles can be redistributed, likely after replenishment through either protein import or fusion with newly synthesized mitochondria (see Fig. 10). Together, our results argue that retrograde transport is critical to the maintenance of these long-lived organelles in neurons.

It is often stated that mitochondria in mature mammalian axons are largely immotile, which may seem incongruous with our data at first glance. However, measures of mitochondrial transport frequency are quite varied and never zero. For example, in cultured rat hippocampal neurons the percent of motile mitochondria range from 20% to 55% over varying time frames, some <5 min (Overly et al., 1996; Ligon and Steward, 2000; Kang et al., 2008; Wang and Schwarz, 2009; Chen and Sheng, 2013; van Spronsen et al., 2013). Using novel *in vivo* imaging methods, Misgeld and colleagues found ∼13% of mitochondria were motile in the exposed sciatic nerve of anesthetized mice, again over the time course of minutes (Misgeld et al., 2007). Therefore, while mitochondria in immature axons do move more frequently (Chang and Reynolds, 2006), these organelles are not completely immobile in mature mammalian axons. In our work, we observe a 50% reduction in mitochondrial occupation of the axon terminal in zebrafish after approximately 2 h, a longer time frame than is used in the aforementioned studies in mammalian neurons. Therefore, our measures of retrograde mitochondrial transport out of axon terminals are within the limits of average mitochondrial transport frequency in axons.

The constant movement of mitochondria in neurons is highly energetically demanding, leading to questions as to why this would be advantageous to the cell. Based on the failed health of mitochondria which cannot move in the retrograde direction, we hypothesize the primary purpose of this movement is organelle repair and maintenance. Mitochondrial maintenance is complicated by the fact that this organelle requires more than a thousand proteins for optimal health and function. While mitochondria maintain their own genome (Schatz et al., 1964; Dawid, 1966; Wolstenholme and Dawid, 1967), which includes genes encoding 13 proteins in humans, the bulk of the >1200 proteins important for the function and maintenance of this organelle are synthesized from genes encoded in the nucleus (Pagliarini et al., 2008; Calvo et al., 2016). These proteins have diverse half-lives, ranging from hours to weeks (Kim et al., 2012; Chan et al., 2015). Once generated, mitochondrial proteins translocate to the correct compartment within the organelle through well-described mitochondrial protein import pathways (Schmidt et al., 2010) or fusion with healthy organelles. However, while we know how proteins are incorporated into the organelle, how proteins are brought to the organelle before import, particularly when the organelle is a long distance from the main source of protein synthesis, the cell body, is largely unknown. Transport of mRNAs to axon terminals has been shown to be sufficient to replenish some mitochondrial proteins (Cioni et al., 2019); however, all mitochondrial proteins using this mechanism would require active transport of over a thousand mRNAs along with their oftentimes disparate translation machinery. In humans, this transport would need to span distances up to ∼1 m from the cell body in humans. Given the number of mitochondrial proteins, the rapid turnover rates of a subset of them, and the distance from the cell body to distal neuronal compartments, active transport of each individual mRNA or protein would be energy intensive (Kim et al., 2012). Alternatively, as our results suggest, mitochondria themselves could be transported from the distal processes toward the cell body for repair.

Maintenance of mitochondrial health and function at sensory and motor axon terminals had differential effects on neural circuit activity in our study. This may be explained by the presynaptic versus postsynaptic nature of these axons. Axonal mitochondria have previously been shown to be essential for local ATP synthesis and calcium buffering to support active synapses at the presynapse, the site of synaptic vesicle release and synaptic vesicle recycling (Werth and Thayer, 1994; Kang et al., 2008). Elegant work on cultured neurons has shown that vesicle cycling at the presynapse utilizes the most ATP during continued stimulation and requires mitochondria for maintenance of presynaptic ATP levels (Rangaraju et al., 2014). Further, work on neuromuscular junctions in *Drosophila* demonstrated that mitochondrial ATP is essential for vesicle recruitment with sustained stimulation (Verstreken et al., 2005). In line with this, in our study, motor neuron axons, which require synaptic vesicle cycling and recruitment at the presynapse, have defects in cytoplasmic ATP levels and synaptic vesicle release as measured by SypHy. Furthermore, we observe deficits in behavioral outputs including spontaneous swim frequency. Conversely, our work in zebrafish pLL sensory axons, which are postsynaptic, do not have any defects in ATP levels or axon terminal activity. As these axons do not need to regulate vesicle cycling and recruitment, it is likely that mitochondria play a much different role in a postsynaptic (sensory) axon terminal. Together, these data indicate that sensory and motor neuron axons are differentially sensitive to defects in mitochondrial health, transport, and function.
